# Population genomic analysis uncovers environmental stress-driven selection and adaptation of *Lentinula edodes* population in China

**DOI:** 10.1038/srep36789

**Published:** 2016-11-10

**Authors:** Yang Xiao, Xuanjin Cheng, Jun Liu, Chuang Li, Wenyan Nong, Yinbing Bian, Man Kit Cheung, Hoi Shan Kwan

**Affiliations:** 1 Institute of Applied Mycology, Huazhong Agricultural University, 430070, Hubei Province, P. R. China; 2 School of Life Sciences, The Chinese University of Hong Kong, Shatin, Hong Kong SAR, P. R. China

## Abstract

The elucidation of genome-wide variations could help reveal aspects of divergence, domestication, and adaptation of edible mushrooms. Here, we resequenced the whole genomes of 39 wild and 21 cultivated strains of Chinese *Lentinula edodes*, the shiitake mushroom. We identified three distinct genetic groups in the Chinese *L. edodes* population with robust differentiation. Results of phylogenetic and population structure analyses suggest that the cultivated strains and most of the wild trains of *L. edodes* in China possess different gene pools and two outlier strains show signatures of hybridization between groups. Eighty-four candidate genes contributing to population divergence were detected in outlier analysis, 18 of which are involved in response to environmental stresses. Gene enrichment analysis of group-specific single nucleotide polymorphisms showed that the cultivated strains were genetically diversified in biological processes related to stress response. As the formation of fruiting bodies is a stress-response process, we postulate that environment factors, such as temperature, drove the population divergence of *L. edodes* in China by natural or artificial selection. We also found phenotypic variations between groups and identified some wild strains that have potential to diversify the genetic pool for improving agricultural traits of *L. edodes* cultivars in China.

The basidiomycete *Lentinula edodes* (Berk.) Singer, Xianggu in Chinese or Shiitake in Japanese, has a wild population distributed in Asia, Australasia and the Americas^1^. *L. edodes* alleviates environmental impacts caused by forest and agricultural wastes as it produces hydrolytic and oxidative enzymes involving in biodegradation of organic substrates^2^. The residues of biodegradation in turn can be used as substrates in mushroom production. Due to its ability to bioconvert, *L. edodes* has been traditionally cultivated to harvest fruiting bodies as superior quality food for human consumption. Additionally, bioactive compounds of *L. edodes* have been reported to have various medicinal properties, such as antivirus, immunomodulation, antitumor, and antioxidation^3^.

*L. edodes* is one of the most popular edible mushrooms worldwide, especially in East Asia. Although *L. edodes* was first cultivated in China more than 800 years ago^4^, little is currently known about its domestication history in China. At the outset of modern cultivation of *L. edodes* in the 1930s, its property of vegetative propagation enabled wild strains to be directly used for cultivation without any genetic improvement^5^. *L. edodes* is a tetrapolar heterothallic basidiomycete^6^, which means that useful traits can be accumulated through crossbreeding between two monokaryons with compatible mating alleles. Compared to the wild strains of *L. edodes*, its cultivars were characterized by a high yield, a high quality of the fruiting body, and precocity. However, the phylogenetic relationship and genetic differentiation between the wild and cultivated strains remain to be elucidated. Moreover, studies of domestication-related genes are important because such genes are pivotal for breeding superior strains.

Efforts have been made to explore the genetic diversity of *L. edodes*^5^,^7^,^8^, However, most studies focused only on either the wild or the cultivated strains, while very few covered a combination of both to illustrate their genetic relationship. Previously, two major groups were revealed among 89 Chinese *L. edodes* cultivars by using inter-simple sequence repeat (ISSR) and sequence-related amplified polymorphism (SRAP) markers^9^. In another study, population structure analysis identified two unambiguous genetic groups among 88 wild strains of Chinese *L. edodes*, which correspond to two geographic regions from which the samples were collected^10^. At present, there are more than 100 *L. edodes* cultivated strains in China but their genetic variation is low. It was postulated that these cultivated strains were derived from a limited number of elite strains^11^.

With the rapid development of genome sequencing, population genomics has been gradually introduced into fungal species including yeasts and some other model organisms^12^,^13^,^14^,^15^. Fungi under domestication were typically selected for their advantageous traits endowed by special genetic loci and they underwent several rounds of population bottlenecks^14^. The molecular evolutionary processes of domestication were successfully elucidated in fungal species such as *Aspergillus* and yeast. *A. oryzae* was domesticated from an atoxigenic lineage of *A. flavus* driven by an extensive remodeling of its metabolism^14^. By comparative genomic analysis, three horizontally transmitted genomic regions that contain genes relevant to wine fermentation were identified to be a domestication fingerprint in wine yeasts^15^. Thus, it is clear that fungal domestication can undergo numerous ways of genomic reorganization.

Hitherto, population genomic analysis has not yet been implemented on commercially cultivated edible mushrooms. Considering the economic and ecological values of edible mushrooms, it is necessary to understand the ecology and evolution of edible mushrooms from a genome-wide perspective. As *L. edodes* was first cultivated in China, we hypothesize that Chinese *L. edodes* cultivars were derived from Chinese wild strains by local domestications and that wild and cultivated strains share the same gene pool. To test this hypothesis, we resequenced 21 cultivated and 39 wild strains of Chinese *L. edodes* and analyzed their genomic and phenotypic variations. The aims of this study were (1) to clarify the genetic diversity and population structure of wild and cultivated strains of Chinese *L. edodes*; (2) to elucidate the genetic relationship and differentiation between wild and cultivated strains; and (3) to investigate the domestication process of Chinese *L. edodes* using methods of population genomics. We revealed the presence of three distinct genetic groups in Chinese *L. edodes*, and showed that the cultivars have gene pools different from most wild strains. Significant differences among most tested phenotypic traits between groups were observed. Furthermore, we identified candidate genes serving in population divergence, especially those for stress response. Our study is a pioneer that sheds light on the genomic variation and population differentiation of edible mushrooms, thereby providing valuable resources for the management and utilization of *L. edodes* in China.

## Materials and Methods

### *Lentinula edodes* strains for sequencing

Sixty *L. edodes* strains from China, including 39 wild and 21 cultivated ones, were used in this study (Supplementary Table S1). In our previous study, the 21 cultivars constituted a core collection of Chinese cultivated strains^9^, while 35 out of the 39 wild strains constituted a core collection of Chinese wild strains. Core collection is a subset of accessions representing the maximum possible genetic diversity contained in the entire collection with minimum repetition^16^. The wild strains were collected from nine provinces, either from natural reserves or mountain areas remote from cultivation sites, or provided by professional research institutes (Supplementary Table S1). The cultivated strains were provided by professional research institutes in six provinces (Supplementary Table S1). Cultivation experimentation was performed at mushroom farms at Huazhong Agricultural University, Wuhan China. The morphology of fruiting bodies indicated that all the tested strains belong to *L. edodes*.

### Genome sequencing, mapping, and genotype calling

The preparation of DNA samples was carried out as previously^10^. Qualified DNA samples were sent to Berry Genomics Co. Ltd (Beijing, China) for library preparation and sequencing, and then they were sequenced on Illumina HiSeq2500 with 125-bp paired-end and a 500-bp insert size. Sequencing reads were trimmed by Trimmomatic Version 0.33^17^ with the default parameters. Adapters, as well as leading and trailing low quality or N bases (below quality 3) in each read, were removed. Reads were scanned with a 4-base wide sliding window and were cut when the average quality per base dropped to below 15. Reads shorter than 36 bases were also dropped.

The L54A reference genome of *L. edodes* consists of 765 scaffolds and 6,334 unplaced contigs (all with lengths above 100 bp) with a total length of 39.8 Mb (GenBank accession number: LOHM00000000). The trimmed reads were aligned to the L54A reference sequences by using the BWA software^18^ with the default settings. The conversion of SAM format to BAM, sorting, indexing, and genotype calling were performed by using the SAMtools software package v1.2^19^. PCR duplicates were removed with Picard version1.119 (http://picard.sourceforge.net), and reads with a minimum mapping quality of 30 and a minimum base quality of 30 (mpileup -q30 -Q30) were kept for analysis. BCFtools (in SAMtools software package) was used to call genotype variants via multiallelic calling (-m) model. The -m option is an alternative model for multiallelic and rare-variant calling. The coefficient of downgrading mapping quality for reads containing excessive mismatches was set to 50 according to the recommendation of the SAMtools manual. With VCFtools v0.1.13^20^, single nucleotide polymorphisms (SNPs) with a quality value below 30 and a mean depth below five were discarded. The statistics of reads after discarding of the PCR duplicates were computed by qualimap v2.1.3^21^. Only bi-allelic SNPs with a minor allele frequency of ≥ 0.05 and without any missing data in the 60 strains were retained for analysis. SNP annotation was performed with the package snpEff version 4.2^22^ based on the L54A reference genome and its annotation.

### Phylogeny and population structure

A neighbor-joining (NJ) tree was constructed using the PHYLIP package v3.69 based on a distance matrix^23^. *Coprinopsis cinerea* was used as the outgroup, and MEGA5^24^ was used to visualize the phylogenetic tree. Principal component analysis (PCA) was performed with SNPRelate on R platform^25^. All filtered SNPs as described above were utilized to compute the NJ tree and PCA plot. The population structure of Chinese *L. edodes* was inferred by the model-based clustering method implemented in ADMIXTURE^26^. In order to decrease the effect of linkage disequilibrium on the ADMIXTURE results, only 19,244 SNPs that were at least 1,000 bp apart in each scaffold or contig were used. Then, we utilized a cross-validation (CV) procedure in ADMIXTURE to estimate the number of the ancestral population (*K*). The *K* value was initially set from 1 to 12. A good value of *K* will exhibit a lower CV error compared to other *K* values. The ADMIXTURE results were plotted by R script.

### Diversity and divergence analyses

Nucleotide diversity (π) and Tajima's *D* within 5-kb non-overlapping genomic windows were calculated with VCFtools at the whole genome level and in genic regions only. We also surveyed the numbers of synonymous and non-synonymous loci in different groups as defined in the NJ tree. The numbers of heterozygous and homozygous loci, and inbreeding coefficients (*F*) of each strain in different groups were investigated by using VCFtools. The divergence between populations was estimated via Weir and Cockerham *F*_*ST*_ by using VCFtools^27^. To compare the genetic variations within and among different populations, Poppr in R was employed to conduct a hierarchical analysis of molecular variance (AMOVA) based on the SNP dataset used in the ADMIXTURE analysis^28^. The total variance was partitioned into variance within individual strains, variance among strains within populations, and variance among populations.

### Genomic signatures of selection

In order to study the natural and the artificial selection of the Chinese *L. edodes* population, we identified outlier SNPs among three groups as defined in the phylogeny analysis by using BayeScan 2.1^29^. BayeScan is designed to identify candidate loci under selection based on the multinomial-Dirichlet model by using differences in allelic frequencies between populations^29^. Furthermore, the Bayesian method takes into account the demographic histories of different populations. The outlier SNPs were detected with the filtering criteria of *q* value <0.05 and *F*_*ST*_ coefficient above the mean by using default settings^30^.

Genomic regions with the highest 5% *F*_*ST*_ value for 5-kb windows between phylogenetic groups were picked as candidate divergent regions. If an outlier SNP identified by BayeScan 2.1 is also located in a 5-kb window with top 5% *F*_*ST*_ value, it is considered as a selection loci for divergence between the two groups. Additionally, the outlier SNPs located in the 5-kb windows with top 5% *F*_*ST*_ value both between Group I and Group II, as well as between Group I and Group III were considered as candidate SNPs related to divergence of Group I. Likewise, we obtained the candidate SNPs related to divergences of Group II and Group III. Furthermore, genes with these outlier SNPs were functionally annotated with the Blast2GO package^31^.

### Functional enrichment analysis

We used in-house scripts to identify group-specific SNPs, *i.e*., SNPs detected in a certain group only, and extracted SNPs annotated as “missense mutation” from these group-specific SNPs. Here, we focused only on missense SNPs because they would alter the amino acid sequences, therefore very likely changing the function of the protein encoded, but such alteration would not be as lethal as a nonsense mutation which results in an incomplete and usually nonfunctional protein. Next, we extracted genes that include these missense SNPs, and these genes were functionally annotated with gene ontology (GO) terms with Blast2GO. Fisher’s exact test in topGO^32^ was implemented to search for enriched GO terms within gene sets.

### Phenotyping

Strains with agronomic traits of fast growth, precocity, good morphologic characteristics of fruiting body, and high yield are prone to be selected during domestication and breeding of *L. edodes*. Thus, we determined 13 such agronomic traits on our tested strains. Mycelium growth rates on both MYG (2% malt extract; 2% glucose; 2% agar; 0.1% peptone; 0.1% yeast extract) medium and mixed sawdust (SD) (DGR-myg and DGR-sd) were measured for all 60 strains by using methods described previously^33^,^34^.

Cultivation experimentation of the core collections, including 21 cultivated and 35 wild strains, were conducted at Huazhong Agricultural University in 2014–2015. The 56 strains were allocated in a mushroom house in accordance with the randomized-block design with two blocks, and four bags of each strain were contained in each replicate. Twenty fruiting bodies from each strain were randomly selected for phenotype determination. Phenotype screening of the 11 fruiting body-related traits was followed as previously reported: FP, the time interval (in days) from incubation to formation of the first primordium (d); FB, the time interval (in days) from incubation to harvest of the first fruiting body (d); PD, pileus diameter (mm); PT, pileus thickness (mm); PW, pileus weight (g); SL, stipe length (mm); SD, stipe diameter (mm); SW, stipe weight (g); NF, number of fruiting bodies (per/bag); WF, weight of a single fruiting body (g); Y, total weight of fruiting bodies per bag (g/bag)^35^. Duncan's multiple range test implemented in SPSS version 17.0 was performed to analyze the difference of these traits between groups.

The value of each phenotype was normalized as follows for heatmap plotting:


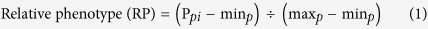


where P_*pi*_ refers to the value of phenotype *p* for individual *i*, min_*p*_ refers to the minimum value of phenotype *p*, and max_*p*_ refers to the maximum value of phenotype *p* across all strains. The heatmap was created with heatmap.2 function in R platform.

## Results

### Statistics of resequencing

We obtained >2 Gb of raw clean data with Q30 > 83% for each strain, while the mean genome coverage of the 60 strains was 88.74% and the mean average depth was 50.25 fold. The mapping rate of reads in different strains varied from 85.01% to 97.63%, with an average of 91.58% (Supplementary Table S1).

### Population structure of *Lentinula edodes* in China

**Figure 1:**
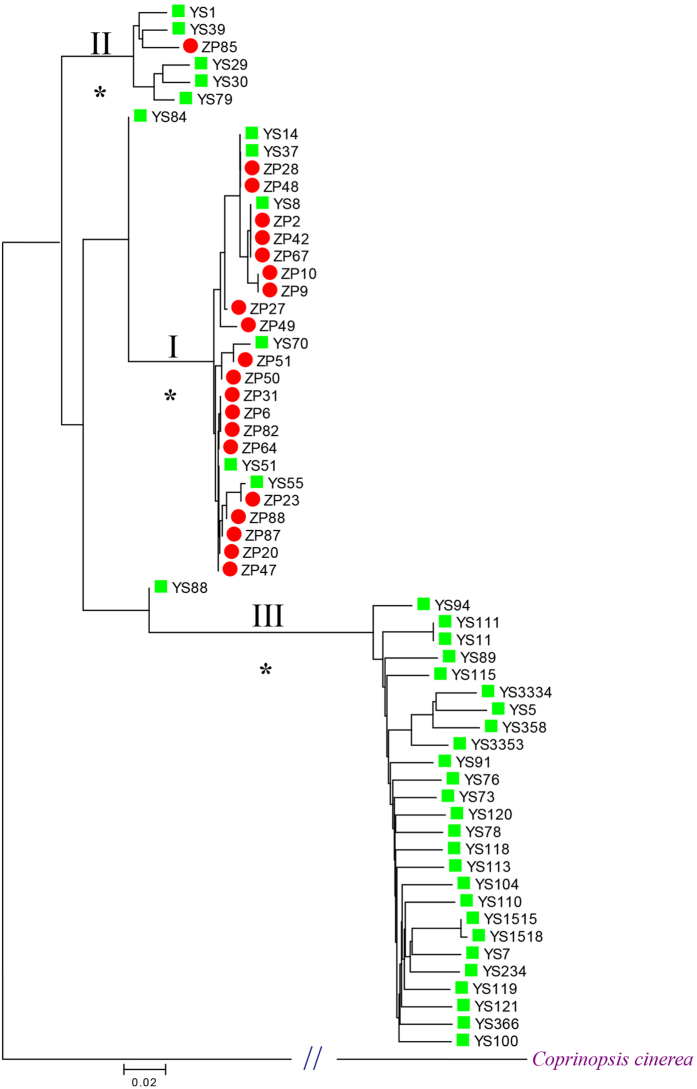
A neighbor-joining phylogenetic tree constructed by using 230,487 SNPs with *Coprinopsis cinerea* as the outgroup. Cultivated strains are marked with 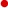 and wild strains are marked with 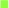. Roman numerals I, II, and III correspond to the three groups detected by phylogenetic analysis. *At nodes denotes bootstrap support values of 100 pseudoreplicates.

**Figure 2:**
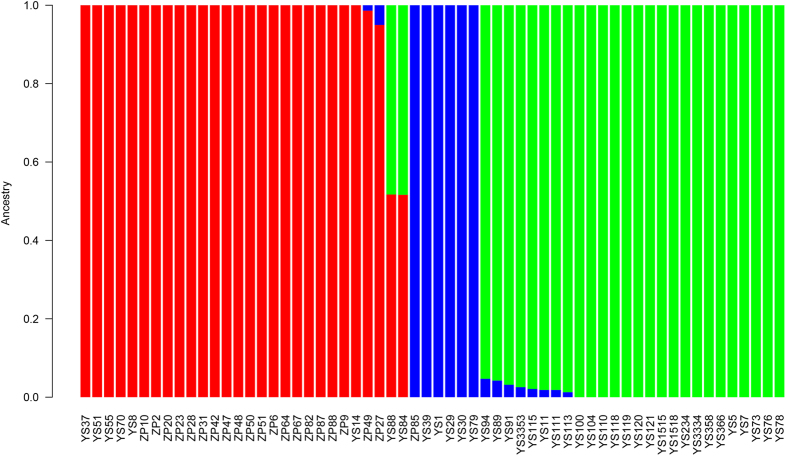
ADMIXTURE results uncovered by 19,244 SNPs (*K* = 3). The distribution of all 60 strains assigned to three groups defined in Fig. 1 is indicated by the color code (Group I: red; Group II: blue; Group III: green). Each strain is represented by a vertical bar, and the y-axis quantifies cluster membership.

After strict filtering, we retained 230,487 SNPs for population genomic analysis. These SNPs were located in 502 scaffolds and 398 contigs (all with lengths above 400 bp), and spanned upon 9,476 gene models. The total length of these scaffolds and contigs was 35.67 Mb, accounting for 89.62% of the whole genome of *L. edodes*. Phylogeny and PCA analyses revealed three distinct groups of Chinese *L. edodes* strains (Fig. 1 and Supplementary Fig. S1). Group I contained 20 cultivated and six wild strains, which was further divided into two subgroups. Group II included five wild and one cultivated strains, while Group III was consisted of 26 wild strains (Fig. 1). Basically, Group I represented a cultivar enriched population, whereas Group II and Group III represented two wild populations. In Group III, all strains except YS11 were collected from Sichuan and Yunnan provinces located in Southwest China (Fig. 1). Furthermore, we used ADMIXTURE to infer individual ancestry and admixture proportions. After CV error estimates, it is clear that *K* = 3 is a sensible modeling choice for our ADMIXTURE analysis (Supplementary Fig. S2). The ADMIXTURE results robustly suggest the presence of three genetic groups among the tested strains (Fig. 2), in congruence with the results of phylogeny analysis and PCA. Two outliers, YS84 and YS88, included mixed ancestries of both Group I and Group III (Fig. 2) and were excluded from downstream between-group analysis.

### Diversity and divergence analyses

The number of SNPs in Group I (88,314), Group II (93,419) and Group III (160,312) account for 38.32%, 40.53%, and 69.55%, respectively, of the total SNPs in the Chinese *L. edodes* population (Table 1). Thus, most of the SNPs were contributed by strains in Group III and this group retained more genetic variation. During domestication, the ratios of intragenic SNPs and total SNPs remained constant, Group I (0.755), Group II (0.769) and Group III (0.775), which were comparable to that in the 60 strains (0.769). The ratios of non-synonymous versus synonymous SNPs were 0.497 (Group I), 0.362 (Group II) and 0.362 (Group III). The higher level of non-synonymous/synonymous in Group I demonstrated that the strains in this group have undergone positive selection.

Similar π values between Group I and Group II indicate that these two groups have comparable levels of genetic variation, both at whole genome level and in genic regions. For all three groups, the π values in the genic regions were slightly lower than those at whole genome level, indicating a lower nucleotide diversity in the genic regions than in the intergenic regions. In addition, the average values of Tajima's *D* were positive in all three groups, suggesting that multiple alleles were present in the Chinese *L. edodes* population with different frequencies.

The numbers of heterozygous loci in different groups were as follows: 43,415 (all 60 strains), 37,571 (Group I), 33,615 (Group II), 48,915 (Group III), and 80,716 (two outlier strains, YS84 and YS88) (Table 2). The two outlier strains had the highest level of heterozygosity followed by Group III, compared to the cultivar enriched Group I (Table 2). The inbreeding coefficient (*F*) of the outlier strains was −0.324, implying an excess of heterozygous loci, which is usually caused by outcrossing. Therefore, the *F* value domesticated signatures of hybridization between groups in the two outlier strains. the *F* value in Group III were 0.198 much less than those in Group I (0.388) and Group II (0.449), displaying a much lower inbreeding level in Group III (Table 2).

The Weir and Cockerham mean *F*_*ST*_ values for 5-kb windows were 0.328 for Group I and Group II, 0.309 for Group I and Group III, and 0.234 for Group II and Group III. When the three groups were considered as a whole, the *F*_*ST*_ value was 0.326, thereby illustrating a relatively high genetic differentiation among the Chinese *L. edodes* population. For the three genetic groups, AMOVA results indicated that the total genetic diversity in *L. edodes* was mainly ascribed to variations within individual strains (59.12%), followed by variations among strains within populations (21.78%), and by variations among populations (19.11%) (Table 3). When all 60 strains were divided into cultivar group and wild group, the AMOVA results were similar to those detected from the three genetic groups. Namely, most of the genetic diversity in *L. edodes* was ascribed to variations within individual strains (55.00%) (Supplementary Table S2).

### Genomic signatures of selection

**Figure 3:**
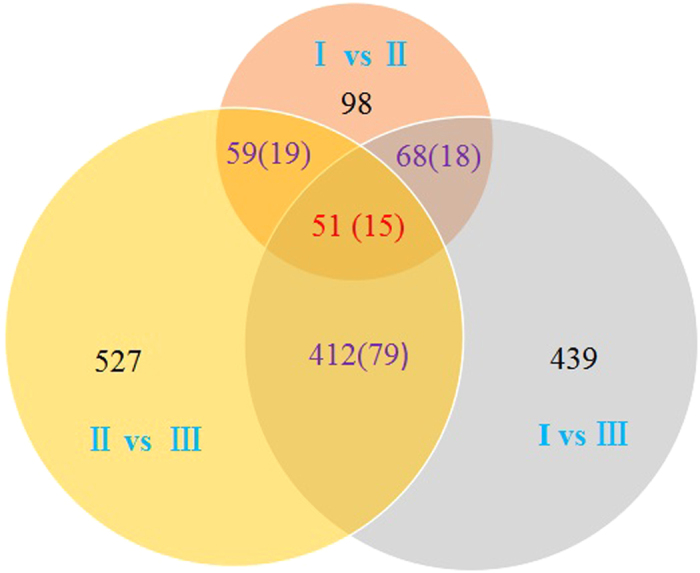
Distributions of 877 outlier SNPs in the 5-kb genomic windows with the highest 5% *F*_*ST*_ value across the three groups defined in Fig. 1. The numbers in brackets represent the numbers of genes that contain the outlier SNPs.

A total of 877 outlier SNPs were detected with BayeScan 2.1. These outlier SNPs were located on 69 scaffolds and four unplaced contigs, spanning over 250 gene models of *L. edodes*. Five hundred and eighty four (66.59%) of the 877 outliers were detected to lie in the 5-kb windows with a top 5% *F*_*ST*_ value, thus suggesting that the majority of the outlier SNPs were related to *L. edodes* population divergence. The numbers of outliers located in the 5-kb windows with the highest 5% *F*_*ST*_ value between all group pairs were 98 (I vs II), 439 (I vs III) and 527 (II vs III) (Fig. 3). Among these, 59 outlier SNPs were shared only by the I-II pair and the II-III pair (Fig. 3), and they could be related to divergence between Group I and Group II, as well as divergence between Group II and Group III. Therefore, these 59 SNPs were considered to be candidate outlier loci related to the divergence of Group II. Similarly, the number of candidate outlier SNPs related to the divergence of Group I was 68 and that of Group III was 412 (Fig. 3). Genes containing these outlier SNPs related to the divergence of a certain group are likely to be candidate genes for population divergence. The corresponding number of genes with these outlier SNPs were 18 for Group I, 19 for Group II, and 79 for Group III. Notably, 15 of these genes were shared between all three groups, *i.e*., these genes were related to the divergence of these three groups. Besides, one gene was shared by Group I and Group II (aug_scv1_leest_g3613), while another was shared by Group I and Group III (aug_scv1_leest_g6391) (Supplementary Table S3).

These 84 genes containing outlier SNPs relevant to population divergence were found to be involved in a wide range of cellular functions, such as kinase activity, chaperone binding, transcription factor activity, and DNA binding (Table 4 and Supplementary Table S3).

### Enriched functions of group-specific missense SNPs

**Figure 4:**
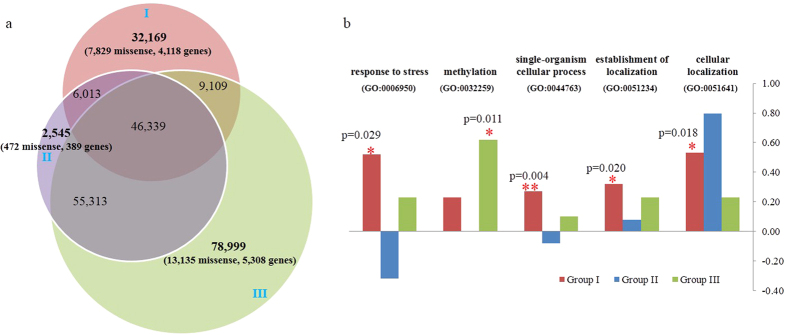
Functional enrichment of group-specific single nucleotide polymorphisms (SNPs) (three groups are defined in Fig. 1). (**a**) Distribution of group-specific SNPs. The numbers in brackets indicate the numbers of group-specific missense SNPs and the genes on which the SNPs are located. (**b**) Log-odds statistics of enriched gene ontology (GO) functions (biological process, level-3). Log-odds for each GO function was calculated as its gene frequency in a group divided by its gene frequency in the whole genome background, then log2-transformed. Positive log-odds plus red stars denote significant enrichment (*p < 0.05; **p < 0.01).

By comparing SNPs between groups, we found that Group III contained the largest collection of unique SNPs (78,999) (i.e. the SNPs present in Group III only). Group I (32,169) followed while Group II (2,545) contained fewest unique SNPs (Fig. 4a). Results from topGO indicated that the missense gene sets of Group III were significantly enriched in “methylation” (22 genes), whereas those of Group I were significantly enriched in four biological processes including “response to stress” (38 genes), “single-organism cellular process” (392 genes), “establishment of localization” (175 genes), and “cellular localization” (44 genes). No enrichment was identified for Group II (Fig. 4b, Supplementary Tables S4–S8).

### Phenotype diversity

**Figure 5:**
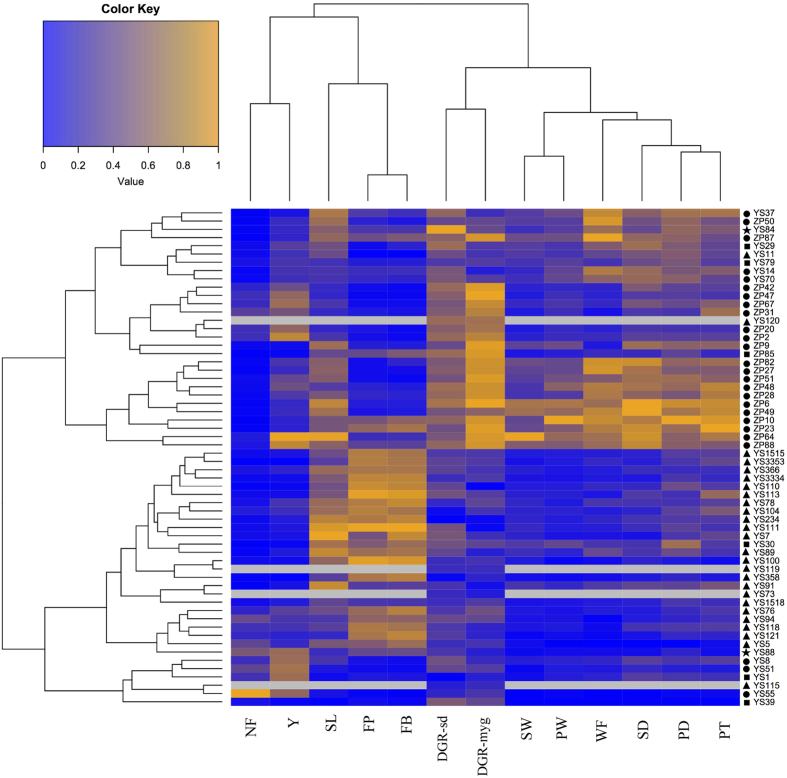
Phenotypic variation of all 60 shiitake strains. The strains are shown in rows and phenotypes in columns. The abbreviation of each phenotype is shown at the bottom: FP, the time interval (in days) from incubation to formation of the first primordium (**d**); FB, the time interval (in days) from incubation to harvest of the first fruiting body (**d**); PD, pileus diameter (mm); PT, pileus thickness (mm); PW, pileus weight (**g**); SL, stipe length (mm); SD, stipe diameter (mm); SW, stipe weight (**g**); NF, number of fruiting bodies (per/bag); WF, weight of a single fruiting body (**g**); Y, total weight of fruiting bodies per bag (g/bag); DGR-myg, mycelium growth rate on MYG medium; DGR-sd, mycelium growth rate on sawdust medium. Phenotype values were normalized according to the color key at the top left corner; missing data are shown in gray shading. Clustering of phenotypes and strains were performed by the complete linkage method with Euclidean distances. The grouping of strains as identified in the phylogenetic analysis are marked in different symbols: ●, Group I; ■, Group II; ▲, Group III; ★, outlier strains.

Phenotypic variations are controlled by genetic variations. To investigate the relationship between genetic structure and phenotypic traits in *L. edodes*, we measured 13 quantitative traits that are most concerned by the shiitake industry (Supplementary Table S9) and analyzed the phenotypic differences between groups. There were significant differences (*P* < 0.05 or *P* < 0.01, Duncan's multiple range test) between groups for all traits except NF and SL, indicating that these quantitative traits were stratified by the population structure (Supplementary Fig. S3). The clustering result of the tested strains by quantitative traits was consistent with that derived from the SNP markers in general (Fig. 5). The strains were grouped into two explicit clusters – one mainly consisted of strains from Group I (21 cultivated and nine wild strains) and another mainly contained strains from Group III (30 wild strains). The relatedness between the 13 traits was clearly presented by clustering. Five traits (NF, Y, FP, FB, and SL) were clustered together, and so was the case for the other eight traits. Among these traits, the closest relationship was identified for the following pairs: NF and Y; FP and FB; DGR-myg and DGR-sd; SW and PW; and PD and PT (Fig. 5).

## Discussion

### Population genomics of cultivated and wild strains

Our study represents a pioneer work on the population genomics of cultivated mushrooms. Results of phylogeny and population structure revealed the presence of three distinct genetic groups across the Chinese *L. edodes* population, with most cultivated strains clustered with six wild strains in Group I and a single cultivated strain among wild strains in Group II. These results showed that cultivars of *L. edodes* in China share two different genetic backgrounds.

Six strains collected from the wild were nested within the cultivar enriched Group I. There are two possible interpretations for this result. First, because *L. edodes* is widely cultivated across China, the spores and mycelia of cultivated strains may easily disperse into the wild either accidentally or by human activities. Thus, these six strains may represent possible cultivars dispersed back into the wild. Second, these six strains may represent true wild strains. As modern cultivation of *L. edodes* was started in the 1930s^5^, the domestication of *L. edodes* is recent. Therefore, these six wild strains and the cultivars may not be differentiated enough to separate into different groups in the phylogenetic analysis. Indeed, the lower *F*_*ST*_ value of 0.150 between two subgroups within Group I indicated a lower genetic differentiation within this group, implying either domestication-associated selection on only a few loci across the genome or the absence of differentiation relevant to cultivation.

If the six strains collected from the wild in Group I are cultivars that recolonized the wild, the whole Group I would represent a complete cultivar group, meaning that the wild strains and a vast majority of cultivars of *L. edodes* in China belonged to different gene pools and there was a domestication event. These suggested two possible scenarios: (1) a strong natural selection in which most cultivated strains adapted to their natural environments, or (2) a robust artificial selection with signatures of domestication. Under the premise that the six strains are cultivars dispersing in the wild, the cultivated strains of *L. edodes* did not seem to derive from any wild strains in China - they formed a distinct clade to most of the wild strains in the phylogenetic tree. It would be possible that the vast majority of Chinese shiitake cultivars were not domesticated from the wild strains in China, at least not from the wild strains we analyzed here, but rather from some other ancestral wild strains. The Japanese strains were introduced into China in the 1960s and the cultivated strains in China are genetically very homogeneous^11^. Some of the Japanese strains, such as the 7401–7405 series and the 79 series, have become main cultivars in China and served as parents for breeding schemes^36^. Although it is possible that the Chinese *L. edodes* cultivars came from Japan, we cannot assume that the parental strains are native to Japan because Japanese breeders widely collect strains all over Asia for *L. edodes* breeding^11^.

On the other hand, if the six strains collected from the wild in group I represent true wild strains, it would mean that the majority of *L. edodes* cultivars in China were domesticated from a few wild strains, perhaps including these six wild strains. Further investigations, such as population genomics with additional strains from both China and other countries such as Japan, may be necessary to uncover the ancestral wild strains that lead to Group I strains.

Considering the geographic origins of the wild strains, 25 out of 26 strains in Group III were collected from Sichuan and Yunnan provinces, and three out of five wild strains in Group II were from two adjacent provinces, Hubei and Hunan (Supplementary Table S1). Therefore, genetic relationships of the wild *L. edodes* strains are highly associated with their geographic distribution, with strains from the same or adjoining regions tending to cluster into the same group. If the six strains represent true wild strains, they should possess the same or adjacent geographic origins. However, the six wild-collected strains in Group I were derived from six different provinces (Supplementary Table S1), and there was no obvious geographic pattern among them. Therefore, it is more likely that the six strains are cultivars dispersed back into the wild.

The cultivated strain ZP85 was located in another clade in the phylogenetic tree as compared with the other cultivars. This is consistent with previous clustering analyses which revealed that ZP85 was distinctly different from other cultivars^7^,^9^. This strain was recently developed from a Chinese wild strain by the Guangdong Institute of Microbiology, and this may be the reason why it was not grouped with the other cultivars.

Two outlier strains (YS84 and YS88) appear to possess mixed ancestry from both wild and cultivated strains. Negative *F* values for both strains indicate that these individuals seem to be hybrids between groups. To verify this, we examined the genotypes of 630 outlier SNPs that are under selection and are located in the genic regions. Fifty-eight strains within Group I, Group II and Group III had homozygous genotypes in the 630 loci, and only these two outlier strains had heterozygous genotypes. Five hundred and eighty-seven out of the 630 loci in YS84 and 601 out of the 630 loci in YS88 were heterozygous genotypes, demonstrating the signatures of hybridization between wild and cultivated groups.

Our results suggest that the cultivated strains of *L. edodes* in China share a different gene pool to the majority of the wild strains. First, the number of SNPs and the nucleotide diversity of the Chinese cultivars were apparently lower than those of the wild strains, which could result from human selection^37^. Second, the genetic differences between the cultivar-enriched group and the wild-strain groups were much larger than those between the two wild groups, as indicated by higher *F*_*ST*_ values (0.328 and 0.309). Genetic differentiation between the two wild-strain groups was also observed, in concordance with a recent report^10^.

### Stress response genes relevant to population divergence

*L. edodes* has been cultivated as a food item with artificial selection and breeding. Almost all phenotypes targeted by artificial selection of *L. edodes* were related to characteristics of fruiting bodies, such as yield, precocity and morphological features. Fruiting body development is often triggered by environmental changes^38^ and the formation of fruiting bodies is believed to be a stress-response process – the fungus senses an unfavorable environment and forms fruiting bodies to disperse its spores to other places for propagation. Environmental factors that lead to fructification in *L. edodes* include temperature fluctuations, nutrient depletion, light and aeration^39^.

Fungi have devised numerous strategies to sense and respond to environmental stresses, including environmental signal transduction pathways, modulation of cytoplasmic membrane fluidity, maintenance of cellular homeostasis, and expression of stress genes^40^,^41^,^42^. Different biomolecules, including proteins, lipids, DNA and RNA, are known to be involved in such processes^41^.

We found that 18 out of the 84 genes relevant to population divergence were linked to fungal stress response and fruiting body initiation. The potential functions of these 18 fungal stress response genes were further described in Supplementary Discussion S1. Fifteen genes relevant to the divergence across all the three groups might be the “master switches” for *L. edodes* population differentiation in China, six of which function in the course of stress response (Table 4 and Supplementary Table S3).

By checking the RNA-seq expression levels (unpublished data) of the 18 genes related to stress response and fruiting body initiation, seven genes were found to be significantly upregulated (>2-fold change) during the transition from mycelium to primordium (Supplementary Fig. S4). Thus, it is possible that these seven genes are related to fruiting body initiation, and stress response-related genes are involved in the biological process of fruiting body initiation. Considering their correlation with population divergence, these genes are candidate genes that deserve further study. Functions of these seven genes during stress response contained signal transduction, transcription initiation, lipid metabolism, and cold or heat response (Table 4).

We also found that Group III-specific variations were significantly enriched in the methylation processes. Most of these methylation processes occured at the DNA level, some at the RNA level and only a few were at the protein level (Supplementary Table S8). Genes in protein methylation involved two enzymes that can modify fungal membrane organization, a sphingolipid C9-methyltransferase (aug_scv1_leest_g7627) and a delta-sterol C-methyltransferase (aug_scv1_leest_g7648). Both sphingolipids and sterols are lipids that constitute the major components of fungal membranes, and they play essential roles in regulating hyphal differentiation^43^. It has been shown that the methylation of sphingolipids and sterols can adjust the rigidity and fluidity of eukaryotic cell membranes^44^. Such modification could enable the shitake strains in Group III to better adapt to their local environments. Meanwhile, methylation at the DNA and RNA levels suggest that there is an upstream gene regulatory network that Group III uses for adaptation. In comparison, Group I-specific variations were significantly enriched in single-organism processes, especially those related to stress response and establishment of localization. These “stress response” genes within Group I-specific variations included some transcriptional regulators, a couple of genes in DNA modification or repair, and several heat shock proteins such as Hsp70, Hsp90, and Hsp40 (Supplementary Table S4). Such a diverse genetic make-up of stress response in Group I reveals a dynamic system that the cultivated strains use to form fruiting bodies facing unfavorable environmental conditions.

Our study has revealed that many genes relevant to population divergence are linked to the response to environmental stresses - a premise to the induction of fruiting body. We postulate that these genes relevant to population divergence are targeted mainly at the fruiting body development stage. Genes encoding kinases, heat-shock proteins and transcription factors function as upstream factors in stress responses. Thus, it is reasonable that modulating the adaptation to the environment and the initiation of fruiting body from the upstream factors would be more effective.

### Environmental drivers for population divergence

Previous studies on fungal population genomics have revealed that adaptation to environmental conditions might be a driver for population divergence^13^,^45^. In *N. crassa*, Ellison *et al*.^13^ found that two genes related to temperature and circadian rhythm in the genomic island of divergence could function in local adaptation^13^. In *Suillus brevipes*, Branco *et al*.^45^ identified that adaptation to the saline environment could motivate recent population divergence; this is because a gene that can enhance salt tolerance in acidic conditions is within an extremely diverged genomic region^45^.

Environmental temperature is crucial for fungal adaptation and population differentiation^46^,^47^,^48^.

Among environmental factors concerning *L. edodes* cultivation, temperature is in particular important. Fruiting is typically induced after mycelial growth by reducing the temperature by at least 5 °C^38^. The induced primordium is unable to develop into fruiting body under constant temperature^49^. The wild strains in Group III were collected from a low-latitude area in Southwest China. In contrast, the wild strains in Group II and the cultivars in Group I were collected mainly from a high-latitude area with a relatively lower temperature. Although the 13 phenotypes examined here were not determined in the original ecological niches where the tested strains were grown, the cultivation test was performed in a mushroom house wherein the environmental conditions were not controlled in a way that mimicked the changing natural conditions. The cultivation test began at the end of August 2014 in Wuhan, central China. The majority of strains in Group I and Group II form primordia and fruiting bodies at lower temperatures in winter, but most of their counterparts in Group III do so in the next spring when the temperature was higher (Supplementary Fig. S3). The phenotypic differences in the temperature response between the groups embodied the genetic variations. Combining the phenotypic differences and the fact that genes related to stress response are under selection, we suggest that the divergence of the Chinese *L. edodes* population is driven by environmental factors, of which temperature is the foremost important one.

### Potential utilization of the wild strains

Our results revealed that the Chinese shiitake cultivars have a relatively low level of genetic diversity, and that some wild strains (YS11, YS29, YS79, and YS84) possess good agricultural traits as deemed desirable by the shiitake industry (Fig. 5). Thus, there is a good potential to improve the current Chinese shiitake cultivars by incorporating genetic resources from wild strains, such as by hybridization. The outlier strain YS84 is a good example of hybrid vigor, and the currently introduced ZP85 is an example of a wild strain that can be used for cultivation (Fig. 5).

In summary, there are three major findings in our study. First, we have revealed that the existing Chinese *L. edodes* cultivars belong to two distinct genetic backgrounds, which are different to most of the Chinese wild strains we tested, and that two outlier strains are possible hybrids between the wild and cultivar groups. Second, we have found that the Chinese *L. edodes* populations diverge in response to environmental stresses and it is quite likely that environmental factors, especially temperature, drove the divergence of the populations. Third, we have demonstrated the potential of introducing wild strains into existing cultivars to diversify their genetic background and to enhance their agricultural traits. Our study is a pioneer that paves the way for further population studies of mushroom species.

## Additional Information

**Accession codes**: The sequence data has been deposited in NCBI Sequence Read Archive (SRA) with the accession number SRP074229.

**How to cite this article**: Xiao, Y. *et al*. Population genomic analysis uncovers environmental stress-driven selection and adaptation of *Lentinula edodes* population in China. *Sci. Rep*. **6**, 36789; doi: 10.1038/srep36789 (2016).

**Publisher’s note:** Springer Nature remains neutral with regard to jurisdictional claims in published maps and institutional affiliations.

## Supplementary Material

Supplementary Information

## Figures and Tables

**Table 1:** Summary of the Chinese *Lentinula edodes* population for the number of total and intragenic SNPs, nucleotide diversity (π) and Tajima's *D* in total and intragenic SNPs, synonymous SNPs and non-synonymous SNPs for 5-kb windows.

	All strains	Group I	Group II	Group III
Total number of SNPs	230,487	88,314	93,419	160,312
Number of intragenic SNPs	177,330	66,658	71,820	124,315
Intragenic SNPs/total SNPs	0.769	0.755	0.769	0.775
Number of non-synonymous SNPs	41,403	17,784	15,272	26,281
Number of synonymous SNPs	99,819	35,804	42,159	72,686
Non-synonymous SNPs/synonymous SNPs	0.415	0.497	0.362	0.362
π (total SNPs)	1.927 × 10^−3^	1.011 × 10^−3^	1.105 × 10^−3^	1.574 × 10^−3^
π (intragenic SNPs)	1.643 × 10^−3^	0.850 × 10^−3^	0.941 × 10^−3^	1.337 × 10^−3^
π (non-synonymous SNPs)	0.393 × 10^−3^	0.271 × 10^−3^	0.253 × 10^−3^	0.319 × 10^−3^
π (synonymous SNPs)	0.971 × 10^−3^	0.491 × 10^−3^	0.580 × 10^−3^	0.811 × 10^−3^
Tajima's *D* (total SNPs)	1.237	1.369	0.418	0.869
Tajima's *D* (intragenic SNPs)	1.116	1.347	0.351	1.013
Tajima's *D* (non-synonymous SNPs)	0.655	0.845	0.219	0.629
Tajima's *D* (synonymous SNPs)	1.106	1.162	0.320	0.944

**Table 2:** The numbers of heterozygous and homozygous loci, and inbreeding coefficients in different groups as defined in the phylogenetic analysis.

Groups	Ne^a^	No^b^	*F*^ c^
All strains	43,415	187,072	0.288
Group I	37,571	192,916	0.388
Group II	33,615	196,872	0.449
Group III	48,915	181,572	0.198
Two outlier strains	80,761	149,726	−0.324

^a^Ne: number of heterozygous loci.

^b^No: number of homozygous loci.

^c^*F*: inbreeding coefficient.

**Table 3:** Analysis of molecular variance (AMOVA) among and within populations of Chinese *Lentinula edodes* strains.

Source	d.f.	SS	MS	VC	% var.
Among populations	2	43533.19	21766.60	551.87	19.11
Among strains within populations	55	163074.95	2965.00	628.87	21.78
Within individual strains	58	99021.00	1707.26	1707.26	59.12
Total	115	305629.14	2657.65	2888.00	100.00

d.f., Degrees of freedom; SS, sum of squared observations; MS, mean of squared observations; VC, variance components; % Var., percentage of total variance.

**Table 4:** Seven stress response genes containing outlier SNPs relevant to population divergence*.

Scaffold	Gene ID	Description	Gene Function	Stress response function reported	Fungal species^#^	References
Le_N7_S166	aug_scv1_leest_g2962^a^	Pbs2-like MAPKK protein	Protein kinase activity; nucleotide binding	Activating Hog1 in the Hog1 -mediated MAPK pathway which is widely involved in resistance to stress	*Candida albicans Heterobasidion annosum*	50, 51, 52
Le_N7_S297	aug_scv1_leest_g6029^a^	DnaJ protein	Heat shock protein binding; ATP binding; response to heat; unfolded protein binding	A cofactor of Hsp70 that is responsible for the initial folding of nascent polypeptide in biotic and abiotic stresses	*Saccharomyces cerevisiae*	53,54
Le_N7_S356	aug_scv1_leest_g7483^b^	SNF2 family DNA-dependent ATPase	ATPase activity; ATP binding; DNA repair; chromatin remolding; helicase activity; response to stress	Interacting with Hsf1 (heat-shock transcription factor) and respond to temperature stress	*S. cerevisiae*	55,56
Le_N7_S438	aug_scv1_leest_g8832^b^	Protein priB	DNA binding; zinc ion binding; RNA polymerase II transcription factor activity	Correlation to fruiting body initiation	*L. edodes*	57
Le_N7_S482	aug_scv1_leest_g9682^b^	Cyclopropane fatty acid synthase	Lipid biosynthetic progress	Taking part in fatty acid metabolism during stress response that triggers the shift from vegetative growth to reproductive growth	*C. cinerea*	58
Le_N7_S488	aug_scv1_leest_g9807^b^	DEAD-box ATP-dependent RNA helicase	Helicase activity; nucleic acid binding; ATP binding; hydrolase activity	Key factors in cold response	*Neurospora crassa*	13
Le_N7_S764	aug_scv1_leest_g12501^b^	DEAD/DEAH box type DNA/RNA helicase	Nucleic acid binding; ATP binding; ATP-dependent helicase activity	Key factors in cold response	*N. crassa*	13

^a^Genes containing outlier SNPs related to the divergences across the three groups.

^b^Genes containing outlier SNPs related to divergence of Group III.

^*^These seven genes have a >2-fold expression change between the mycelium and primordium stages in shiitake strain L54.

^#^Gene functions involved in stress response are reported in these fungal species.
