# Supplementary material for: Population genomic analysis uncovers environmental stress-driven selection and adaptation of Lentinula edodes population in China
Source: Sci Rep. 2016 Nov 10;6:36789. doi: 10.1038/srep36789 (PMC5103288; doi:10.1038/srep36789)
Supplement: Supplementary Information [file srep36789-s1.pdf]

Title: Population genomic analysis uncovers environmental stress-driven selection and adaptation of *Lentinula edodes* population in China

Authors: Yang Xiao[1,2], Xuanjin Cheng[2], Jun Liu[1], Chuang Li[1], Wenyan Nong[2], Yinbing Bian[1], Man Kit Cheung[2], Hoi Shan Kwan[2]

Affiliations: [1] Institute of Applied Mycology, Huazhong Agricultural University, 430070, Hubei Province, P. R. China

[2] School of Life Sciences, The Chinese University of Hong Kong, Shatin, Hong Kong SAR, P. R. China

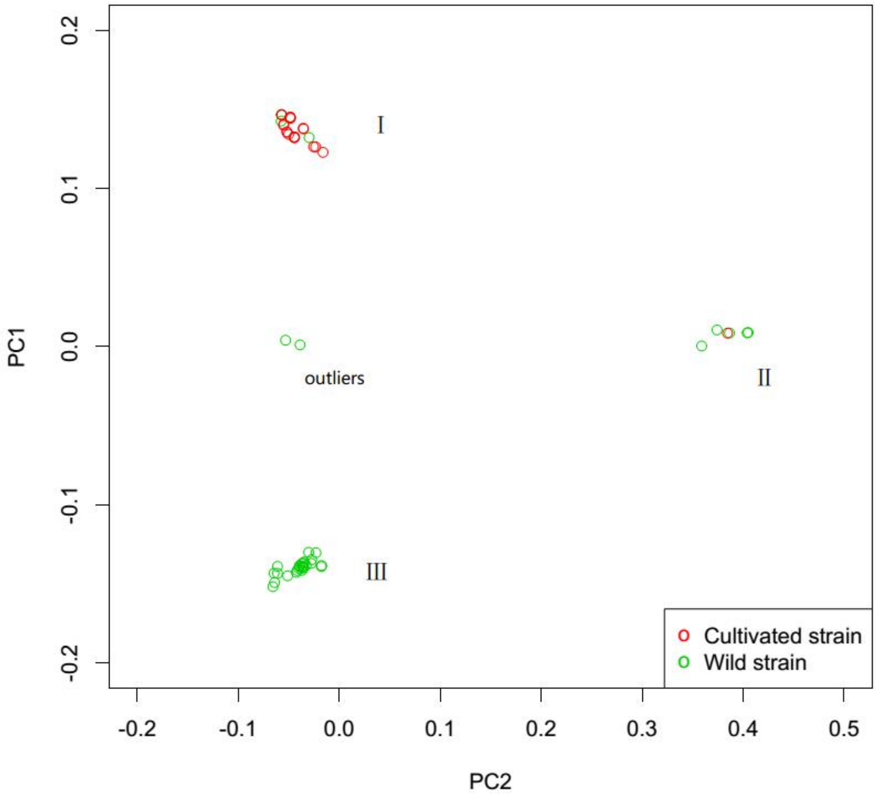

Supplementary Figure S1. Principal component analysis (PCA) using genome-wide SNPs. The group names correspond to those in Figure 1.

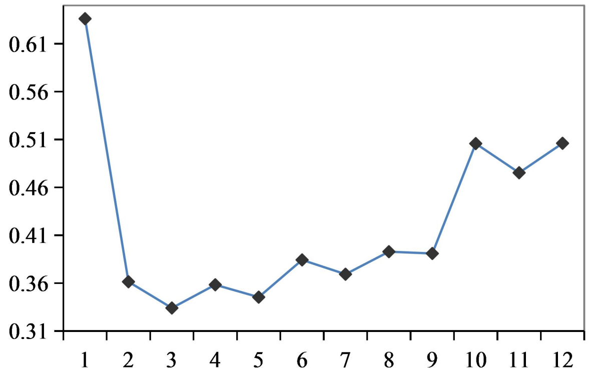

Supplementary Figure S2. Cross-validation plot for the SNP dataset when *K* was set from 1 to 12. A good value of *K* will exhibit a lower cross-validation error compared to other *K* values.

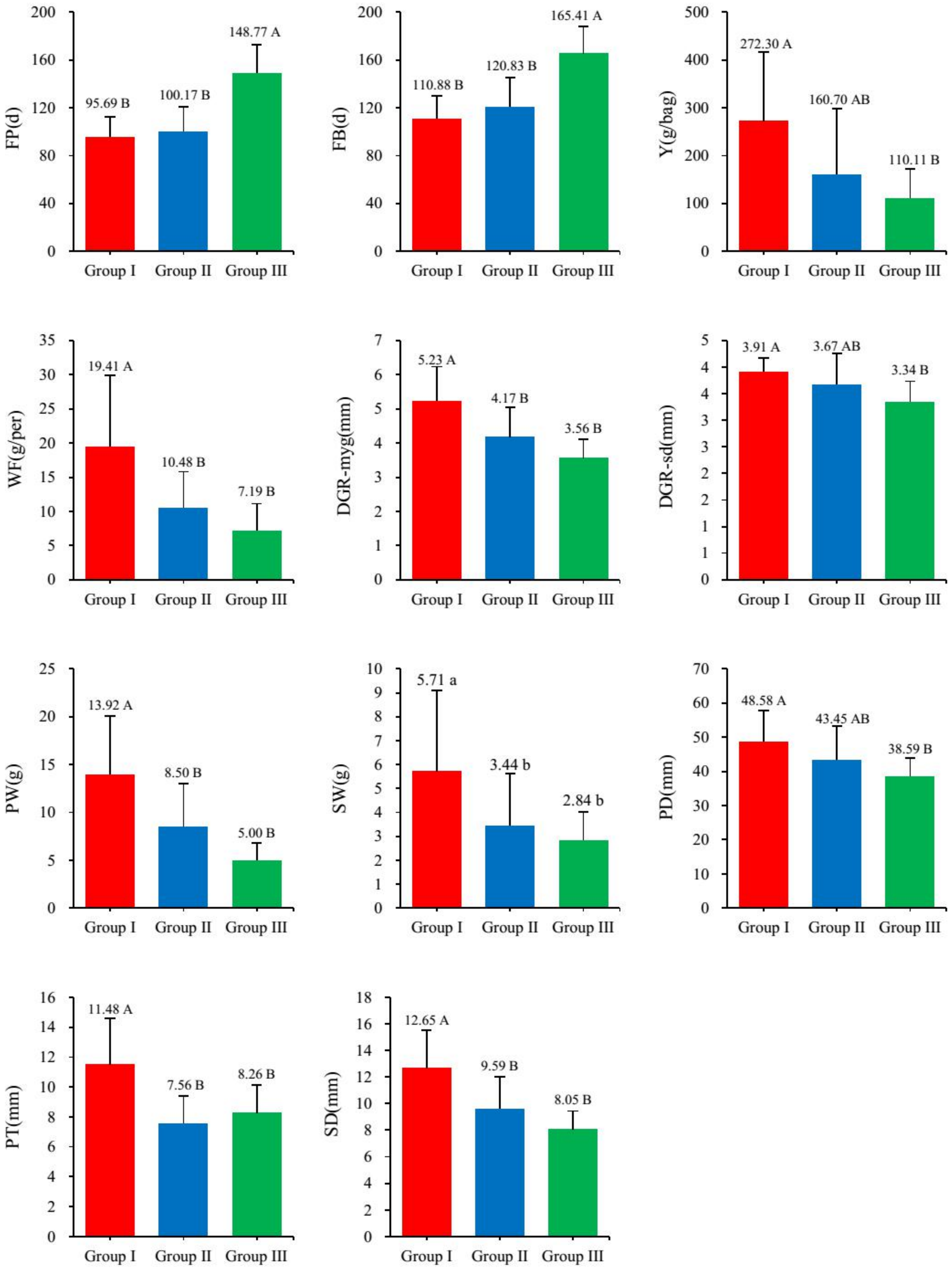

Supplementary Figure S3. Eleven phenotypic traits that were significantly different between groups defined in Figure 1. Numbers above the column chart indicate the means of each trait in the three groups. Significant differences at 0.01 level (uppercase letters) are indicated by different letters by using the Duncan multiple comparison, while that at 0.05 level is displayed in lowercase letters. The 11 phenotypic traits are as follows: FP, the time interval (in days) from incubation to formation of the first primordium (d); FB, the time

interval (in days) from incubation to harvest of the first fruiting body (d); PD, pileus diameter (mm); PT, pileus thickness (mm); PW, pileus weight (g); SD, stipe diameter (mm); SW, stipe weight (g); WF, weight of a single fruiting body (g); Y, total weight of fruiting bodies per bag (g/bag); DGR-myg, mycelium growth rate on MYG medium; DGR-sd, mycelium growth rate on sawdust medium.

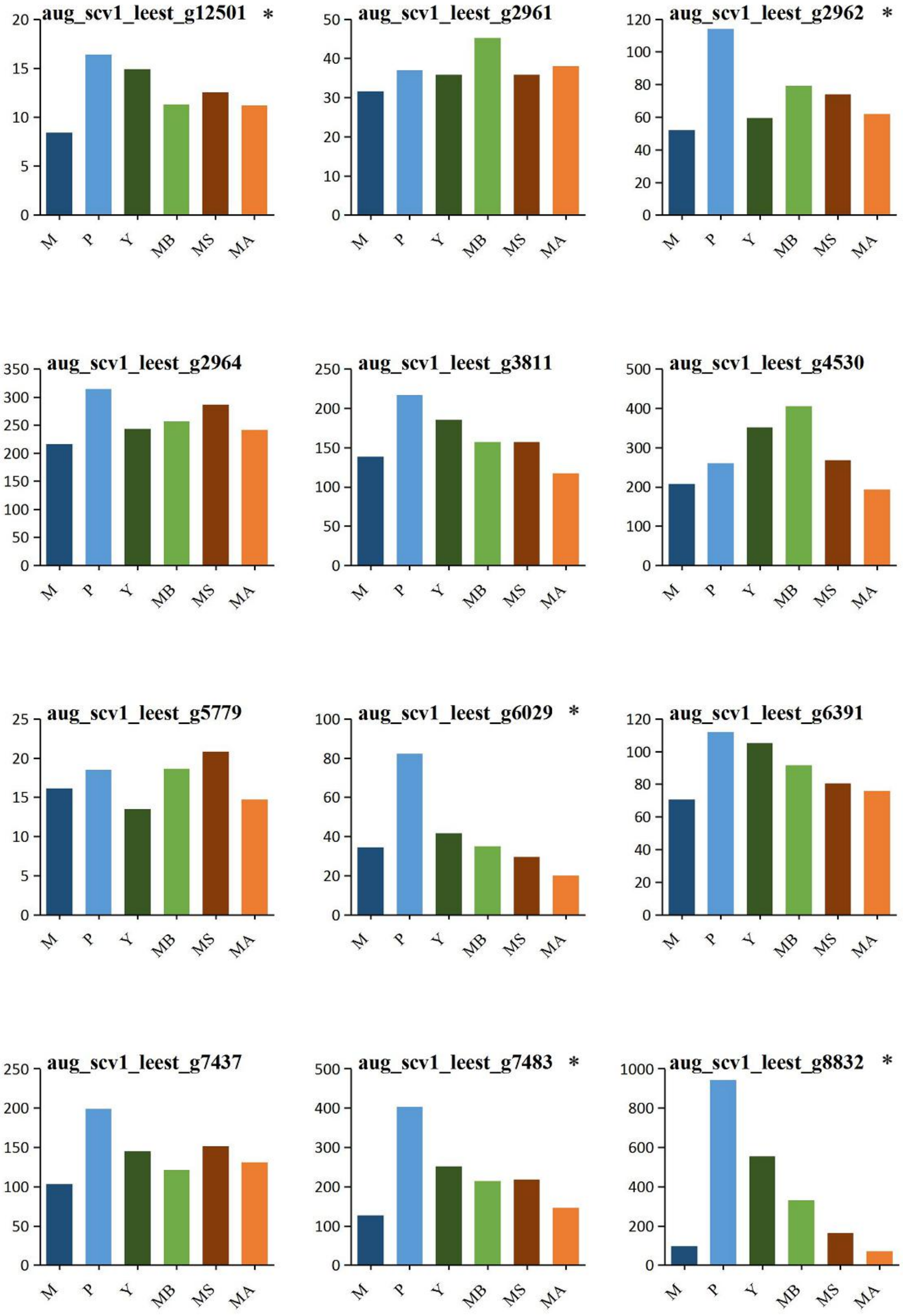
aug_scv1_leest_g12501 *
aug_scv1_leest_g2961
aug_scv1_leest_g2962 *
aug_scv1_leest_g2964
aug_scv1_leest_g3811
aug_scv1_leest_g4530
aug_scv1_leest_g5779
aug_scv1_leest_g6029 *
aug_scv1_leest_g6391
aug_scv1_leest_g7437
aug_scv1_leest_g7483 *
aug_scv1_leest_g8832 *
M
P
Y
MB
MS
MA

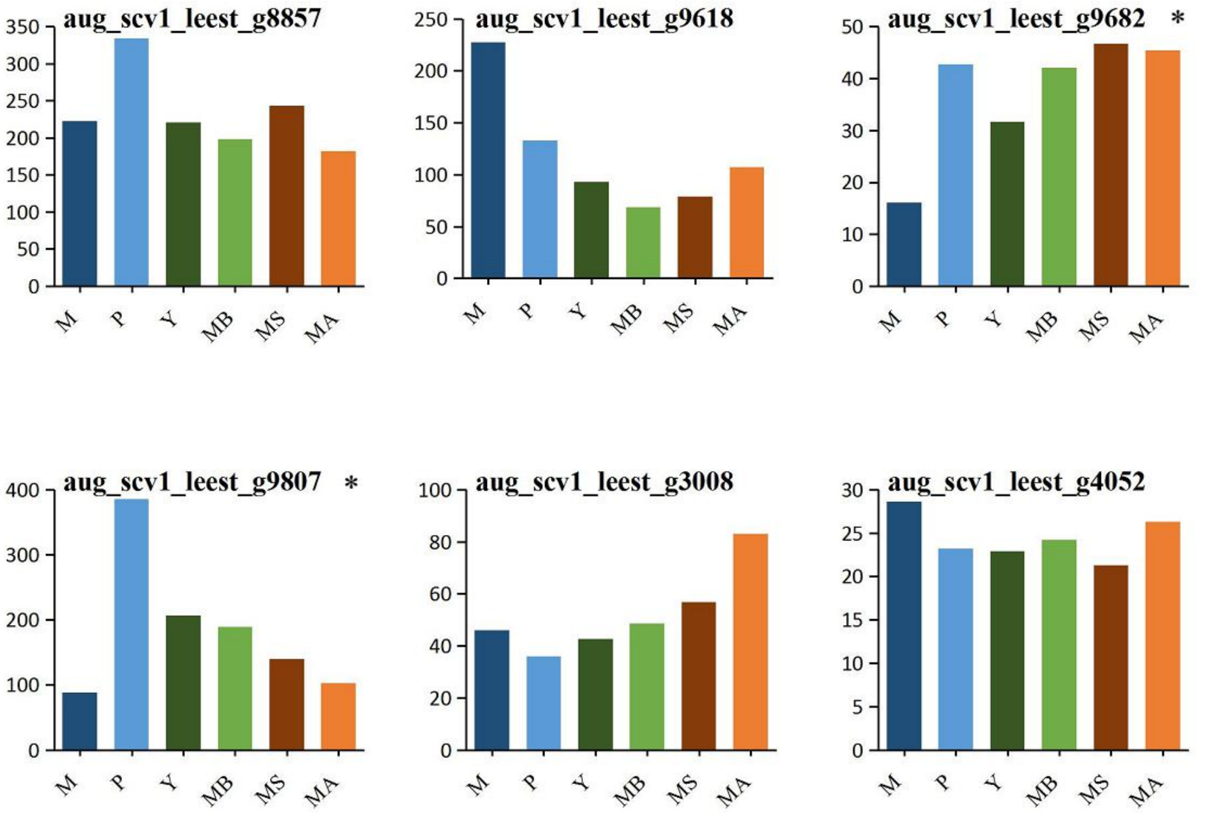

Supplementary Figure S4. Expression levels of the 18 genes under selection related to stress response in shiitake strain L54. These are from our unpublished RNA-seq data. Gene expression levels were normalized by RPKM (reads per kilo base per million mapped reads). M: L54 Mycelium incubated in 12 hours light/12 hours dark; P: Primordium; Y: Young fruiting body; MB: Mature fruiting body before sporulation; MS: Mature fruiting body during sporulation; MA: Mature fruiting body after sporulation. * Genes with a > 2-fold expression change between M and P stages.

Supplementary Table S1 Summary of *Lentinula edodes* strains and statistics of reads for each strain

Strain	Strain type	Strain name	Origin (Province)*	Reads($\times10^6$)	Mapped Reads ($\times10^6$)	Mapped rate (%)	Genome coverage(%)	Average depth (×)
YS1	wild	EFISAAS0229	Guizhou	17.45	16.17	92.68	87.38	45.2
YS100	wild	LMLHA18	Sichuan	24.93	22.38	89.78	86.68	61.46
YS104	wild	LMLHA36	Sichuan	17.87	16.04	89.75	86.37	44.42
YS11	wild	ACCC50786	Anhui	16.81	14.97	89.08	86.42	41.91
YS110	wild	LMLHL22	Sichuan	35.39	31.77	89.78	86.56	87.76
YS111	wild	LMLHL26	Sichuan	30.16	26.74	88.65	86.9	73.94
YS113	wild	LMLHL210	Sichuan	17.75	15.91	89.65	86.46	44.26
YS115	wild	LMYN13	Sichuan	21.57	19.07	88.39	88.19	53.3
YS118	wild	LMYP62	Sichuan	17.50	15.55	88.86	86.79	43.17
YS119	wild	LPZH211	Sichuan	20.36	18.12	88.98	86.45	50.25
YS120	wild	LPZH315	Sichuan	20.29	18.29	90.14	86.1	50.5
YS121	wild	LPG82	Sichuan	37.15	33.03	88.9	87.03	90.8
YS14	wild	GAN059	Gansu	16.35	14.94	91.37	89.04	42.41
YS1515	wild	YAASSM1515	Yunnan	17.79	15.81	88.87	86.73	44.14
YS1518	wild	YAASSM1518	Yunnan	27.85	24.81	89.1	86.39	68.49
YS234	wild	YAASSM234	Yunnan	25.87	23.08	89.21	86.93	63.28
YS29	wild	HUB039	Hubei	15.92	14.52	91.19	87.82	41.59
YS30	wild	HUB040	Hubei	18.89	17.19	91.01	87.58	48.52
YS3334	wild	YAASSM3334	Yunnan	15.01	12.76	85.01	86.22	35.5
YS3353	wild	YAASSM3353	Yunnan	15.58	14.11	90.58	86.3	39.72
YS358	wild	YAASSM358	Yunnan	17.47	15.42	88.23	86.23	43.05
YS366	wild	YAASSM366	Yunnan	33.82	30.58	90.41	86.78	84.37

YS37	wild	HUB091	Hubei	17.16	15.8	92.08	88.59	45.16
YS39	wild	HN002	Hunan	15.46	14.09	91.1	87.72	39.65
YS5	wild	EFISAAS0351	Yunnan	15.89	14.3	89.98	85.6	39.85
YS51	wild	SHX044	Shanxi	18.63	18.06	96.93	93.54	51.82
YS55	wild	NO.41	Jiangxi	17.80	16.71	93.88	88.55	48.02
YS7	wild	00167	Yunnan	23.71	20.83	87.84	86.93	57.71
YS70	wild	LeQc743s	Sichuan	16.54	15.73	95.07	89.91	45.5
YS73	wild	LeQs7211	Sichuan	15.70	13.75	87.58	87.01	38.06
YS76	wild	LeWs735	Sichuan	15.09	13.15	87.18	86.74	36.6
YS78	wild	LHLy14	Sichuan	17.61	15.67	89.02	86.33	43.62
YS79	wild	LHLy217	Sichuan	17.18	15.82	92.04	89.18	44.48
YS8	wild	00168	Yunnan	16.47	15.42	93.61	88.49	43.64
YS84	wild	LMLH14	Sichuan	17.04	15.67	91.94	89.61	44.26
YS88	wild	LMLH36	Sichuan	20.07	18.05	89.94	91.63	50.74
YS89	wild	LMLH52	Sichuan	18.75	16.77	89.44	87.04	46.88
YS91	wild	LMLH59	Sichuan	16.62	14.9	89.67	86.53	41.52
YS94	wild	LMLH116	Sichuan	17.82	15.96	89.55	87.94	44.9
ZP10	cultivated	L135	Fujian	15.70	14.68	93.52	88.74	41.78
ZP2	cultivated	S605	Shanghai	20.79	19.45	93.56	88.5	55.92
ZP20	cultivated	L856	Fujian	15.42	15.03	97.46	93.34	42.65
ZP23	cultivated	S602	Shanghai	16.67	15.81	94.83	89.89	45.13
ZP27	cultivated	Guangxiang-51	Guangdong	16.45	15.26	92.7	91.17	43.28
ZP28	cultivated	Hunong-1	Shanghai	16.43	15.12	92.02	88.68	42.97
ZP31	cultivated	Huaxiang-8	Hubei	24.70	23.68	95.88	93.59	67.16
ZP42	cultivated	Qingke-20	Zhejiang	14.71	13.61	92.54	88.37	37.94
ZP47	cultivated	Qiu-6	Hubei	18.76	18.11	96.57	93.55	52.03

ZP48	cultivated	Rifen-34	Henan	15.92	14.63	91.91	88.91	42.46
ZP49	cultivated	Senyuan-10	Hubei	24.79	23.34	94.15	90.09	65.24
ZP50	cultivated	Senyuan-1	Hubei	18.86	17.75	94.11	92.17	51.61
ZP51	cultivated	Senyuan-2	Hubei	16.96	16.06	94.68	91.98	46.69
ZP6	cultivated	Cr04	Fujian	16.05	15.35	95.68	93.54	44.04
ZP64	cultivated	430	Hubei	15.85	15.48	97.63	93.47	44.03
ZP67	cultivated	908	Henan	16.28	15.23	93.59	88.62	43.32
ZP82	cultivated	868	Zhejiang	17.28	16.45	95.21	93.53	47.15
ZP85	cultivated	Xiangjiu	Guangdong	28.55	26.23	91.88	87.63	73.11
ZP87	cultivated	Yuhua-2	Henan	27.67	25.61	92.54	93.58	72.44
ZP88	cultivated	Yuhua-4	Henan	18.76	18.08	96.24	93.48	52.06
ZP9	cultivated	L12	Fujian	17.00	15.54	91.41	88.85	43.54

* origins of the cultivated strains are the provinces where the affiliations providing these strains are located.

Supplementary Table S2 Analysis of molecular variance (AMOVA) among and within populations when all tested strains were divided into cultivar group and wild group

Source	d.f.	SS	MS	VC	% var.
Among populations	1	58802.17	58802.17	1029.22	31.99
Among strains within populations	58	151194.32	2606.80	418.62	13.01
Within individual strains	60	106173.50	1769.56	1769.56	55.00
Total	119	316170.00	2656.90	3217.40	100.00

d.f., Degree of freedom; SS, sum of squared observations; MS, mean of squared observations; VC, variance components; % Var., percentage of total variance.

Supplementary Table S3 Seventy-seven genes containing outlier SNPs relevant to population divergence

Scaffold	Gene ID	Description	Gene Function
Le_N7_S166	aug_scv1_leest_g2961[a] *	BAG domain-containing protein	Chaperone binding; ADP binding;microtubule motor activity
Le_N7_S166	aug_scv1_leest_g2964[a] *	Kinase	Nucleotide binding; protein kinase activity
Le_N7_S189	aug_scv1_leest_g3811[a] *	Glycerol-3-phosphate o-acyltransferase	Transferase activity; transferring acyl groups
Le_N7_S222	aug_scv1_leest_g4530[a] *	C4-methyl sterol oxidase	Iron ion binding; oxidoreductase activity; oxidation-reduction process; fatty acid biosynthetic process
Le_N7_S311	aug_scv1_leest_g6391[b,d] *	Tuberin	Regulation of small GTPase mediated signal transduction; GTPase activator activity; TSC1-TSC2 complex;negative regulation of TOR signaling; positive regulation of GTPase activity
Le_N7_S166	aug_scv1_leest_g3008[c] *	Endoplasmic oxidoreductin-1	Protein disulfide isomease activity; oxidoreductase activity
Le_N7_S201	aug_scv1_leest_g4052[c] *	Nuclear distribution PAC1	Dynein binding; hydrolase activity
Le_N7_S279	aug_scv1_leest_g5779[d] *	Atypical/RIO/RIO1 protein kinase	ATP binding; protein serine/threonine kinase

			activity
Le_N7_S356	aug_scv1_leest_g7437[d] *	Gef1	Rho guanyl-nucleotide exchange factor activity
Le_N7_S438	aug_scv1_leest_g8857[d] *	CMGC/DYRK/DYRK2 protein kinase	Protein kinase activity;ATP binding
Le_N7_S476	aug_scv1_leest_g9618[d] *	GATA-4 5 6 transcription factor	Sequence-specific DNA binding transcription factor activity;zinc ion binding
Le_N7_S160	aug_scv1_leest_g2794[a]	Predicted protein	
Le_N7_S166	aug_scv1_leest_g2959[a]	WD repeat-containing protein	Protein binding
Le_N7_S166	aug_scv1_leest_g2963[a]	BRCT-containing protein	
Le_N7_S222	aug_scv1_leest_g4513[a]	Predicted protein	
Le_N7_S222	aug_scv1_leest_g4516[a]	Predicted protein	
Le_N7_S409	aug_scv1_leest_g8421[a]	Activating signal cointegrator 1 complex subunit 1	
Le_N7_S554	aug_scv1_leest_g10689[a]	Translation initiation factor eif-2b subunit alpha	Cellular metabolic process
Le_N7_S557	aug_scv1_leest_g10735[a]	Poly(A) RNA polymerase cid1	Nucleotide transferase activity
Le_N7_S557	aug_scv1_leest_g10741[a]	Dipeptidyl-peptidase III	Dipeptidyl-peptidase activity; proteolysis
Le_N7_S179	aug_scv1_leest_g3613 [b,c]	Transcriptional activator acu-15	DNA binding; zinc ion binding
Le_N7_S298	aug_scv1_leest_g6131[b]	Type IV secretion protein Rhs	
Le_N7_S166	aug_scv1_leest_g3009[c]	Predicted protein	
Le_N7_S19	aug_scv1_leest_g90[d]	Hypothetical protein	
Le_N7_S23	aug_scv1_leest_g182[d]	20S proteasome subunit	Threonine-type endopetidase activity
Le_N7_S102	aug_scv1_leest_g1713[d]	Hypothetical protein	
Le_N7_S140	aug_scv1_leest_g2413[d]	DNA repair rad5	Nucleic acid binding; ATP bingding; zinc ion binding; hydrolase activity
Le_N7_S160	aug_scv1_leest_g2798[d]	DUF625 domain	
Le_N7_S160	aug_scv1_leest_g2800[d]	Mitochondrial import receptor subunit tom20	Protein targeting
Le_N7_S160	aug_scv1_leest_g2801[d]	Mitochondrial import receptor subunit tom20	Protein binding
Le_N7_S166	aug_scv1_leest_g2958[d]	Homeobox HD-10	Organic compound bingding; heterocyclic compound binding
Le_N7_S187	aug_scv1_leest_g3761[d]	u4 u6 small nuclear ribonucleo	mRNA splicing via spliceosome
Le_N7_S189	aug_scv1_leest_g3810[d]	Hypothetical protein	Zinc ion binding; RNA

			polymerase II transcription factor activity
Le_N7_S201	aug_scv1_leest_g4034[d]	Glycoside hydrolase family 31	carbohydrate binding;hydrolase activity
Le_N7_S201	aug_scv1_leest_g4035[d]	WD40 repeat	Protein binding
Le_N7_S218	aug_scv1_leest_g4359[d]	Alpha/beta hydrolase fold protein	
Le_N7_S222	aug_scv1_leest_g4476[d]	Peroxisomal biogenesis factor 6	ATP binding
Le_N7_S222	aug_scv1_leest_g4477[d]	Gar1-domain-containing protein	Ribosome biogenesis
Le_N7_S222	aug_scv1_leest_g4478[d]	Cell cycle control protein CDC50	
Le_N7_S222	aug_scv1_leest_g4479[d]	Clathrin adaptor, mu subunit	Protein transport
Le_N7_S222	aug_scv1_leest_g4480[d]	An1-type zinc finger 1	Zinc ion binding
Le_N7_S222	aug_scv1_leest_g4481[d]	Vacuolar sorting DID4	Vacuolar transport
Le_N7_S222	aug_scv1_leest_g4482[d]	COG4-domain-containing protein	
Le_N7_S222	aug_scv1_leest_g4532[d]	Sulfate permease	transporter activity
Le_N7_S225	aug_scv1_leest_g4564[d]	Uncharacterized protein	DNA binding
Le_N7_S225	aug_scv1_leest_g4580[d]	malate dehydrogenase	malate dehydrogenase activity
Le_N7_S225	aug_scv1_leest_g4643[d]	Vacuolar ATP synthase	ATP binding; hydrolase activity
Le_N7_S279	aug_scv1_leest_g5780[d]	Dihydroxy-acid dehydratase	Catalytic activity
Le_N7_S297	aug_scv1_leest_g6039[d]	Proteasome activator complex subunit 4	Integral component of membrance;binding
Le_N7_S317	aug_scv1_leest_g6462[d]	/	
Le_N7_S317	aug_scv1_leest_g6463[d]	Zinc finger protein	Metal binding;nucleic acid binding
Le_N7_S356	aug_scv1_leest_g7398[d]	Hypothetical protein	
Le_N7_S356	aug_scv1_leest_g7399[d]	SPRY-domain-containing , partial	Protein binding
Le_N7_S356	aug_scv1_leest_g7411[d]	Acyl- N-acyltransferase	N-acetyltransferase activity
Le_N7_S356	aug_scv1_leest_g7484[d]	Cytochrome P450	Iron ion binding;oxidoreductase activity;oxidation-reduction process;heme binding
Le_N7_S438	aug_scv1_leest_g8839[d]	Cysteine-type endopeptidase	Peptidase activity
Le_N7_S438	aug_scv1_leest_g8854[d]	Ribosomal protein L15	Structural molecule activity
Le_N7_S438	aug_scv1_leest_g8855[d]	Pre-mRNA splicing factor 38A	
Le_N7_S438	aug_scv1_leest_g8856[d]	Clr6 histone deacetylase associated phd -2 cph2	Protein binding;zinc ion binding
Le_N7_S443	aug_scv1_leest_g8987[d]	P-loop containing nucleoside triphosphate hydrolase	Nucleic acid binding;ATP binding
Le_N7_S457	aug_scv1_leest_g9085[d]	Delta2-dienoyl- isomerase	Catalytic activity
Le_N7_S476	aug_scv1_leest_g9616[d]	Hypothetical protein	Transcription factor activity; zinc ion binding;

			sequence-specific DNA binding
Le_N7_S482	aug_scv1_leest_g9683[d]	GTP-binding protein adp-ribosylation factor-like protein 2	GTP binding
Le_N7_S482	aug_scv1_leest_g9684[d]	Eukaryotic translation initiation factor 5B	Amino acid transmembrance transporter activity;GTPase activity
Le_N7_S482	aug_scv1_leest_g9696[d]	Proteophosphoglycan ppg4	RNA polymerase II transcription cofactor activity
Le_N7_S482	aug_scv1_leest_g9698[d]	Rab escort	Oxidoreductase activity
Le_N7_S488	aug_scv1_leest_g9808[d]	Proteophosphoglycan ppg4	Sequence-specific DNA binding;sequence-specific DNA binding transcription factor activity
Le_N7_S554	aug_scv1_leest_g10687[d]	Methionine aminopeptidase	Metal ion binding;aminopeptidase activity
Le_N7_S631	aug_scv1_leest_g11253d	Cytochrome c oxidase subunit 3	Cytochrome-c oxidase activity;transporter activity
Le_N7_S635	aug_scv1_leest_g11302[d]	Ribosomal protein S10	Structural constituent of ribosome
Le_N7_S635	aug_scv1_leest_g11303[d]	Deoxyribonuclease TATDN2	Endodeoxyribonuclease activity
Le_N7_S635	aug_scv1_leest_g11304[d]	GPI ethanolamine phosphate transferase 3, partial	Catalytic activity;transferase activity
Le_N7_S649	aug_scv1_leest_g11494[d]	Major facilitator superfamily MFS-1	transporter activity
Le_N7_S764	aug_scv1_leest_g12469[d]	Hypothetical protein	DNA binding
Le_N7_S764	aug_scv1_leest_g12471[d]	Hypothetical protein	DNA binding; zinc ion binding; RNA polymerase II transcription factor activity
Le_N7_S764	aug_scv1_leest_g12502[d]	P-loop containing nucleoside triphosphate hydrolase protein	DNA binding;ATP binding;hydrolase activity
Le_N7_S764	aug_scv1_leest_g12503[d]	Chromatin assembly factor subunit	

[*] Stress response genes containing outlier SNPs

[a] Genes containing outlier SNPs related to the divergences across the three groups.

[b] Genes containing outlier SNPs related to divergence of Group I.

[c] Genes containing outlier SNPs related to divergence of Group II.

[d] Genes containing outlier SNPs related to divergence of Group III.

Supplementary Table S4 Gene set enriched in stress response in Group I specific SNPs

Gene ID	Description	Molecule function	Cellular component	Biologic process
aug_scv1_leest_g1023	transcription factor Tfb2		nucleus	regulation of biological process;response to stress;DNA metabolic process
aug_scv1_leest_g10615	heat-shock protein 90	nucleotide binding;protein binding		protein metabolic process;response to stress
aug_scv1_leest_g11146	PREDICTED: Werner syndrome ATP-dependent helicase homolog	nucleic acid binding;exonuclease activity;3'-5' exonuclease activity;protein binding	intracellular;nucleus	nucleobase-containing compound metabolic process
aug_scv1_leest_g11305	RecName: Full=Flap endonuclease 1; Short=FEN-1; AltName: Full=Flap structure-specific endonuclease 1	nuclease activity;DNA binding;binding	mitochondrion;nucleolus;nucleoplasm	response to stress;DNA metabolic process;biosynthetic process;catabolic process
aug_scv1_leest_g11713	heat shock protein 70	nucleotide binding		response to stress
aug_scv1_leest_g12367	DNA helicase	hydrolase activity		DNA metabolic process
aug_scv1_leest_g12432	DNA repair-related protein	metal ion binding;zinc ion binding		
aug_scv1_leest_g1454	condensin complex subunit SMC1	nucleotide binding	chromosome;nucleus	organelle organization;cell cycle;DNA metabolic process;response to stress
aug_scv1_leest_g1634	DNA repair helicase	DNA binding;hydrolase activity;nucleotide	nucleus	response to stress;DNA

		binding		metabolic process
aug_scv1_leest_g1759	MutS protein homolog 4	nucleotide binding;DNA binding		response to stress;DNA metabolic process
aug_scv1_leest_g1995	regulator of filamentous growth and virulence Rfg1	DNA binding;sequence-specific DNA binding transcription factor activity		symbiosis, encompassing mutualism through parasitism;response to biotic stimulus;response to external stimulus;cell communication;response to stress;regulation of biological process
aug_scv1_leest_g2285	DnaJ-domain-containing protein	binding;protein binding		protein metabolic process
aug_scv1_leest_g2358	RecName: Full=Double-strand break repair protein MRE11	nuclease activity;binding	nucleus	cell cycle;response to stress;DNA metabolic process
aug_scv1_leest_g2827	DnaJ-domain-containing protein	binding;protein binding;nucleotide binding		protein metabolic process;response to stress;response to abiotic stimulus
aug_scv1_leest_g3209	cytochrome c peroxidase	binding;catalytic activity;antioxidant activity		response to stress;metabolic process
aug_scv1_leest_g367	DNA glycosylase	nucleic acid binding;catalytic activity		response to stress;DNA metabolic process
aug_scv1_leest_g4253	putative heat shock transcription factor	sequence-specific DNA binding transcription factor activity;DNA binding	nucleus	response to stress;regulation of biological process

aug_scv1_leest_g4296	DNA ligase	nucleotide binding;binding;catalytic activity		response to stress;DNA metabolic process
aug_scv1_leest_g5043	DNA helicase	DNA binding;hydrolase activity;nucleotide binding		response to stress;DNA metabolic process
aug_scv1_leest_g6047	DNA repair protein	DNA binding;hydrolase activity;nucleotide binding	nucleus	lipid metabolic process;response to stress;DNA metabolic process
aug_scv1_leest_g6173	activator of Hsp90 ATPase	ATPase activator activity;chaperone binding		positive regulation of ATPase activity;response to stress
aug_scv1_leest_g6508	DNA binding protein	nucleotide binding;DNA binding	nucleus	response to stress;DNA metabolic process
aug_scv1_leest_g656	patatin-domain-containing protein	binding;hydrolase activity;antioxidant activity;catalytic activity		metabolic process;lipid metabolic process;response to stress
aug_scv1_leest_g6728	heat shock protein 70	nucleotide binding		response to stress
aug_scv1_leest_g6929	TFIIH basal transcription factor complex subunit SSL1	binding	nucleus	response to stress;DNA metabolic process;regulation of biological process
aug_scv1_leest_g7063	Rad51-associated protein Brh2			response to stress;DNA metabolic process
aug_scv1_leest_g7284	DNA ligase I	DNA binding;nucleotide binding;catalytic activity		DNA metabolic process;biosynthetic process;response to

				stress
aug_scv1_leest_g7483	SNF2 family DNA-dependent ATPase	nucleic acid binding;hydrolase activity;nucleotide binding		response to stress
aug_scv1_leest_g7719	manganese dependent peroxidase 1	binding;catalytic activity;antioxidant activity		response to stress;catabolic process
aug_scv1_leest_g7943	uracil-DNA glycosylase	hydrolase activity		response to stress;DNA metabolic process
aug_scv1_leest_g7997	PIN domain-like protein	nuclease activity;DNA binding	nucleus	response to stress;DNA metabolic process
aug_scv1_leest_g8102	DNA polymerase lambda	DNA-directed DNA polymerase activity;DNA binding		nucleotide-excision repair
aug_scv1_leest_g822	SWR1-complex protein 4			biological_process
aug_scv1_leest_g8908	DNA-(apurinic or apyrimidinic site) lyase	nuclease activity;DNA binding	intracellular	response to stress;DNA metabolic process
aug_scv1_leest_g8930	DNA glycosylase	catalytic activity		response to stress;DNA metabolic process
aug_scv1_leest_g9203	heat shock protein Hsp90	binding		
aug_scv1_leest_g9587	XPG I-region protein	nuclease activity		DNA repair;nucleic acid phosphodiester bond hydrolysis
aug_scv1_leest_g9710	methylated-DNA--cysteine S-met	catalytic activity;methylated-DNA-[protein]-cysteine S-methyltransferase activity		DNA repair

Supplementary Table S5 Gene set enriched in cellular localization in Group I specific SNPs

Gene ID	Description	Molecule function	Cellular component	Biologic process
aug_scv1_leest_g10073	related to Mitochondrial DnaJ chaperone	protein binding		
aug_scv1_leest_g1019	AousoA		cytoplasm;cell	transport
aug_scv1_leest_g10392	vacuolar assembling/sorting protein VPS16		cytoplasm	intracellular protein transport
aug_scv1_leest_g10665	Crm1-F1	transporter activity	protein complex;nuclear envelope	protein transport
aug_scv1_leest_g10737	karyopherin Kap95	transporter activity	protein complex;nuclear envelope	protein transport
aug_scv1_leest_g11767	gamma-adaptin	transporter activity	Golgi apparatus;cytoplasm;protein complex	transport;protein transport
aug_scv1_leest_g1179	Sec1-like snare protein			
aug_scv1_leest_g11817	coatomer complex protein	structural molecule activity	cytoplasmic membrane-bounded vesicle;Golgi apparatus;protein complex	transport
aug_scv1_leest_g11839	VHS domain-containing protein		intracellular	protein transport
aug_scv1_leest_g12463	related to Beta3 protein (Ruby)		cytoplasm;protein complex	transport;protein transport
aug_scv1_leest_g1580	transport protein particle complex subunit		intracellular	transport
aug_scv1_leest_g1762	vacuolar sorting-associated protein Vps27	binding	intracellular	protein transport
aug_scv1_leest_g1821	Adaptor protein complex AP-3 delta subunit	transporter activity	cytoplasm;protein complex;Golgi apparatus;cell;endosome	protein transport;transport;biol ogical_process;cell

				differentiation;regulation of biological process;multicellular organismal development;cellular component organization
aug_scv1_leest_g2035	Sec1-like protein			transport
aug_scv1_leest_g2138	coatomer subunit alpha-2	structural molecule activity	Golgi apparatus;protein complex;cytoplasmic membrane-bounded vesicle	transport;protein transport
aug_scv1_leest_g2165	translation initiation factor 6	translation factor activity, nucleic acid binding;binding		translation;organelle organization
aug_scv1_leest_g2396	sec24-like protein			transport;protein transport
aug_scv1_leest_g2424	ATP binding protein			transport
aug_scv1_leest_g2982	related to Cutl1 or CASP protein		Golgi apparatus	transport
aug_scv1_leest_g3057	expressed protein		protein complex;nuclear envelope	transport
aug_scv1_leest_g4041	alpha/beta hydrolase family domain-containing protein	hydrolase activity	endoplasmic reticulum	lipid metabolic process;protein transport
aug_scv1_leest_g5702	DigA protein	binding	intracellular	transport;protein transport
aug_scv1_leest_g5841	DUF1692-domain-containing protein			
aug_scv1_leest_g6432	endoplasmic reticulum-derived transport			transport

	vesicle ERV46			
aug_scv1_leest_g6433	vacuolar protein sorting-associated protein 45			transport
aug_scv1_leest_g6743	uncharacterized protein		intracellular	protein transport
aug_scv1_leest_g7013	CPII coat sec24 protein	binding	cytoplasmic membrane-bounded vesicle;Golgi apparatus;protein complex	protein transport;transport
aug_scv1_leest_g7086	V-snare-domain-containing protein		Golgi apparatus;cell	transport;protein transport
aug_scv1_leest_g7255	vesicular-fusion protein SEC17		cell;intracellular	transport;protein transport
aug_scv1_leest_g7418	vesicle-mediated transport-related protein			protein transport
aug_scv1_leest_g7622	archain 1		cytoplasm;protein complex	transport;protein transport
aug_scv1_leest_g7656	coatomer protein			transport
aug_scv1_leest_g7757	ARM repeat-containing protein	transporter activity	nuclear envelope;protein complex	protein transport
aug_scv1_leest_g7859	t-SNARE	protein binding	cell;intracellular	transport;protein transport
aug_scv1_leest_g8004	DnaJ domain-containing protein	protein binding		
aug_scv1_leest_g8339	clathrin heavy chain 1	structural molecule activity	cytoplasm;protein complex;plasma membrane;cytoplasmic membrane-bounded vesicle;Golgi apparatus	transport;protein transport
aug_scv1_leest_g849	exocyst complex protein		cytoplasm	transport

aug_scv1_leest_g8493	ran/spi1 binding protein	protein binding	cytoplasm	protein metabolic process;catabolic process;protein transport;transport;cell cycle
aug_scv1_leest_g8841	SDA1-domain-containing protein			
aug_scv1_leest_g8895	transport protein particle complex subunit		Golgi apparatus;organelle	transport
aug_scv1_leest_g8971	signal recognition particle protein	RNA binding;hydrolase activity;nucleotide binding	cytoplasm	nucleobase-containing compound metabolic process;catabolic process;protein transport
aug_scv1_leest_g9269	signal recognition particle binding protein	binding;nucleotide binding;hydrolase activity	endoplasmic reticulum;protein complex	nucleobase-containing compound metabolic process;catabolic process;protein transport
aug_scv1_leest_g96	coatomer protein	structural molecule activity	Golgi apparatus;protein complex;cytoplasmic membrane-bounded vesicle	transport;protein transport
aug_scv1_leest_g9700	importin alpha re-exporter	transporter activity	protein complex;nuclear envelope	protein transport

Supplementary Table S6 Gene set enriched in establishment of localization in Group I specific SNPs

Gene ID	Description	Molecule function	Cellular component	Biologic process
aug_scv1_leest_g10002	ion transport domain-containing protein	ion channel activity	cell	ion transport;transport
aug_scv1_leest_g10003	MFS general substrate transporter		cell	transport
aug_scv1_leest_g10073	related to Mitochondrial DnaJ chaperone	protein binding		
aug_scv1_leest_g1019	AousoA		cytoplasm;cell	transport
aug_scv1_leest_g10291	Sodium/sulfate symporter			
aug_scv1_leest_g10371	multidrug resistance protein 4		cell	transport
aug_scv1_leest_g10392	vacuolar assembling/sorting protein VPS16		cytoplasm	intracellular protein transport
aug_scv1_leest_g10665	Crm1-F1	transporter activity	protein complex;nuclear envelope	protein transport
aug_scv1_leest_g10713	Ca-transporting ATPase	hydrolase activity;transporter activity;binding;nucleotide binding	cell	ion transport;nucleobase-containing compound metabolic process;biosynthetic process
aug_scv1_leest_g10737	karyopherin Kap95	transporter activity	protein complex;nuclear envelope	protein transport
aug_scv1_leest_g11002	OPT oligopeptide transporter			transmembrane transport
aug_scv1_leest_g11145	CDF-like metal transporter	transporter activity	cell	ion transport;transport
aug_scv1_leest_g1117	ammonium transporter	transporter activity	cell	
aug_scv1_leest_g11209	ABC transporter	hydrolase activity;nucleotide binding		biological_process;transport
aug_scv1_leest_g11232	NTF2-like protein		intracellular	transport

aug_scv1_leest_g1138	P-loop containing nucleoside triphosphate hydrolase protein	hydrolase activity;nucleotide binding		biological_process;transport
aug_scv1_leest_g11384	sodium/hydrogen exchanger	transporter activity	cell	transport;biological_process;ion transport
aug_scv1_leest_g11388	snare-like protein	transferase activity	organelle;intracellular;cytoplasm;cell	cellular component organization;transport;organelle organization
aug_scv1_leest_g11408	MFS general substrate transporter			
aug_scv1_leest_g11410	MFS general substrate transporter		cell	transport
aug_scv1_leest_g11436	protein transporter SEC13		cell;organelle;cytoplasm;nucleus;endoplasmic reticulum;intracellular	biological_process
aug_scv1_leest_g11443	MFS general substrate transporter		cell	transport
aug_scv1_leest_g11447	OPT oligopeptide transporter			transport
aug_scv1_leest_g11536	putative sugar transporter protein			transport
aug_scv1_leest_g11767	gamma-adaptin	transporter activity	Golgi apparatus;cytoplasm;protein complex	transport;protein transport
aug_scv1_leest_g1179	Sec1-like snare protein			
aug_scv1_leest_g11798	Cu-transporting P-type ATPase	binding;hydrolase activity;transporter activity;nucleotide binding	cell	ion transport;nucleobase-containing compound metabolic process;biosynthetic process
aug_scv1_leest_g11817	coatomer complex protein	structural molecule activity	cytoplasmic membrane-bounded vesicle;Golgi apparatus;protein complex	transport

aug_scv1_leest_g11829	CDF-like metal transporter	transporter activity	cell	ion transport;transport
aug_scv1_leest_g11839	VHS domain-containing protein		intracellular	protein transport
aug_scv1_leest_g11964	voltage-dependent ion-selective channel	ion channel activity	mitochondrion	transport;regulation of biological process;ion transport
aug_scv1_leest_g12009	membrane transporter		integral to membrane	transmembrane transport
aug_scv1_leest_g12253	MFS general substrate transporter	transporter activity	cell	transport
aug_scv1_leest_g12267	vacuolar assembling protein VPS41	zinc ion binding	intracellular	vesicle-mediated transport;intracellular protein transport
aug_scv1_leest_g12286	protein transporter	binding;transporter activity;nucleotide binding;hydrolase activity	cell	
aug_scv1_leest_g12299	putative mfs transporter protein		cell	transport
aug_scv1_leest_g12429	Voltage-gated hydrogen channel 1	ion channel activity	integral to membrane;membrane	transmembrane transport;ion transport;transport
aug_scv1_leest_g1244	amino acid transporter	transporter activity	cell	transport
aug_scv1_leest_g12463	related to Beta3 protein (Ruby)		cytoplasm;protein complex	transport;protein transport
aug_scv1_leest_g1505	voltage-gated potassium channel beta-2 subunit	ion channel activity	cell	ion transport;transport
aug_scv1_leest_g1509	MFS general substrate transporter		cell	transport
aug_scv1_leest_g1580	transport protein particle complex subunit		intracellular	transport

aug_scv1_leest_g1762	vacuolar sorting-associated protein Vps27	binding	intracellular	protein transport
aug_scv1_leest_g1821	Adaptor protein complex AP-3 delta subunit	transporter activity	cytoplasm;protein complex;Golgi apparatus;cell;endosome	protein transport;transport;biological_process;cell differentiation;regulation of biological process;multicellular organismal development;cellular component organization
aug_scv1_leest_g1985	signal transducing adaptor protein STAM/STAM2		intracellular	protein transport
aug_scv1_leest_g2035	Sec1-like protein			transport
aug_scv1_leest_g2138	coatomer subunit alpha-2	structural molecule activity	Golgi apparatus;protein complex;cytoplasmic membrane-bounded vesicle	transport;protein transport
aug_scv1_leest_g2148	multidrug resistance-associated ABC transporter	hydrolase activity;nucleotide binding		biological_process;transport
aug_scv1_leest_g2165	translation initiation factor 6	translation factor activity, nucleic acid binding;binding		translation;organelle organization
aug_scv1_leest_g2204	voltage-gated potassium channel			transport
aug_scv1_leest_g2332	urea transporter		cell	transport
aug_scv1_leest_g2396	sec24-like protein			transport;protein transport
aug_scv1_leest_g2424	ATP binding protein			transport
aug_scv1_leest_g245	OPT oligopeptide transporter	transporter activity		transport
aug_scv1_leest_g2452	expressed protein	transporter activity	cytoplasm;cell	transport

aug_scv1_leest_g246	OPT oligopeptide transporter	transporter activity		transport
aug_scv1_leest_g2511	potassium:hydrogen antiporter			transport
aug_scv1_leest_g2610	unnamed protein product	transporter activity	cell	transport
aug_scv1_leest_g2643	adrenoleukodystrophy protein	hydrolase activity;nucleotide binding	cell	transport;nucleobase-containing compound metabolic process;catabolic process
aug_scv1_leest_g2702	scamp-domain-containing protein		cell	protein transport
aug_scv1_leest_g2752	calcium transporting ATPase	hydrolase activity;transporter activity;nucleotide binding;binding	cell;Golgi apparatus	transport;cellular component organization;protein transport;organelle organization;ion transport
aug_scv1_leest_g2810	general substrate transporter	transporter activity	cell	transport
aug_scv1_leest_g2833	ARM repeat-containing protein	RNA binding	cytoplasm;nucleus	transport;nucleobase-containing compound metabolic process
aug_scv1_leest_g2914	P-type ATPase	binding;hydrolase activity;transporter activity;nucleotide binding	cell	nucleobase-containing compound metabolic process;catabolic process;ion transport
aug_scv1_leest_g2982	related to Cutl1 or CASP protein		Golgi apparatus	transport
aug_scv1_leest_g3057	expressed protein		protein complex;nuclear envelope	transport
aug_scv1_leest_g3262	ATP18 subunit J of the mitochondrial F1F0 ATP synthase	transporter activity	intracellular;protein complex	generation of precursor metabolites and energy;nucleobase-containing compound metabolic process;ion transport;biosynthetic process

aug_scv1_leest_g3263	vacuolar membrane protein	binding		transport
aug_scv1_leest_g3318	APC amino acid permease	transporter activity	cell	transport
aug_scv1_leest_g3376	cytosine-purine permease	transporter activity	cell	transport
aug_scv1_leest_g3742	L-methionine transporter	transporter activity	cell	transport
aug_scv1_leest_g3743	copper chaperone TahA	binding		ion transport
aug_scv1_leest_g3904	phospholipid-translocating ATPase	binding;transporter activity;nucleotide binding;hydrolase activity	cell	transport
aug_scv1_leest_g3914	mitochondrial NAD transporter		cell	transport
aug_scv1_leest_g3942	Major facilitator superfamily		cell	transport
aug_scv1_leest_g4041	alpha/beta hydrolase family domain-containing protein	hydrolase activity	endoplasmic reticulum	lipid metabolic process;protein transport
aug_scv1_leest_g4060	MFS general substrate transporter		cell	transport
aug_scv1_leest_g4077	f-box protein pof6		cytoplasm	transport
aug_scv1_leest_g4153	APC amino acid permease		cell	transport
aug_scv1_leest_g4186	protein transport protein Sec22		cell	transport
aug_scv1_leest_g4391	MFS general substrate transporter		cell	transport
aug_scv1_leest_g4673	amino acid transporter		cell	transport
aug_scv1_leest_g4685	P-loop containing nucleoside triphosphate hydrolase protein	hydrolase activity;transporter activity;nucleotide binding	cell	transport;nucleobase-containing compound metabolic process;catabolic process

aug_scv1_leest_g4725	pleiotropic drug resistance ABC transporter	hydrolase activity;transporter activity;nucleotide binding	cell	transport;nucleobase-containing compound metabolic process;catabolic process
aug_scv1_leest_g4731	putative hexose transporter protein	transporter activity	cell	transport
aug_scv1_leest_g4748	ATP synthase E chain domain-containing protein	transporter activity	protein complex;mitochondrion	generation of precursor metabolites and energy;nucleobase-containing compound metabolic process;ion transport;biosynthetic process
aug_scv1_leest_g4904	mitochondrial carrier		cell	transport
aug_scv1_leest_g4985	voltage-gated potassium channel beta-2 subunit			transport
aug_scv1_leest_g5083	Not1-domain-containing protein	transporter activity	cell	transport
aug_scv1_leest_g512	GTP-binding protein ypt5	nucleotide binding		protein transport;signal transduction
aug_scv1_leest_g5202	voltage-gated potassium channel beta-2 subunit	ion channel activity	cell	ion transport;transport
aug_scv1_leest_g5221	sodium-hydrogen antiporter			transport
aug_scv1_leest_g5544	OPT oligopeptide transporter			transport
aug_scv1_leest_g560	MFS general substrate transporter		cell	transport
aug_scv1_leest_g5679	carnitine/acyl carnitine carrier		cell	transport
aug_scv1_leest_g5695	rab GTPase activator	enzyme regulator activity		protein transport
aug_scv1_leest_g5702	DigA protein	binding	intracellular	transport;protein transport
aug_scv1_leest_g5766	transthyretin			transport
aug_scv1_leest_g5841	DUF1692-domain-containing			

	protein			
aug_scv1_leest_g597	nucleobase transmembrane transporter	nucleobase transmembrane transporter activity	membrane	nucleobase transport
aug_scv1_leest_g598	nucleobase transmembrane transporter	transporter activity	cell	transport
aug_scv1_leest_g5991	MFS general substrate transporter		cell	transport
aug_scv1_leest_g6028	MFS general substrate transporter	transporter activity	cell	transport
aug_scv1_leest_g6094	ATPase V1 complex subunit C, partial	hydrolase activity;transporter activity	cell;protein complex	ion transport
aug_scv1_leest_g6161	MFS general substrate transporter		cell	transport
aug_scv1_leest_g6179	MFS general substrate transporter		cell	transport
aug_scv1_leest_g6275	oligopeptide transporter			transport
aug_scv1_leest_g6307	general substrate transporter	transporter activity	cell	transport
aug_scv1_leest_g6380	putative alpha-ketoglutarate-dependent taurine dioxygenase protein	catalytic activity		metabolic process
aug_scv1_leest_g6432	endoplasmic reticulum-derived transport vesicle ERV46			transport
aug_scv1_leest_g6433	vacuolar protein sorting-associated protein 45			transport

aug_scv1_leest_g6734	general substrate transporter	transporter activity	cell	transport
aug_scv1_leest_g6743	uncharacterized protein		intracellular	protein transport
aug_scv1_leest_g6779	NicO-domain-containing protein	metal ion binding	integral to membrane	transmembrane transport;metal ion transport
aug_scv1_leest_g6828	MFS general substrate transporter		cell	transport
aug_scv1_leest_g6844	related to lactose permease	transporter activity	cell	transport
aug_scv1_leest_g6922	mitochondrial iron ion transporter	transporter activity	cell	ion transport;transport
aug_scv1_leest_g6942	DUF805 domain membrane protein	transporter activity	cell	ion transport
aug_scv1_leest_g6949	clc channel	ion channel activity	cell	
aug_scv1_leest_g6953	mitochondrial carrier		cell	transport
aug_scv1_leest_g6997	high-affinity cell membrane calcium channel	ion channel activity	cell	ion transport;transport
aug_scv1_leest_g7013	CPII coat sec24 protein	binding	cytoplasmic membrane-bounded vesicle;Golgi apparatus;protein complex	protein transport;transport
aug_scv1_leest_g7086	V-snare-domain-containing protein		Golgi apparatus;cell	transport;protein transport
aug_scv1_leest_g7202	mRNA transport regulator	protein transporter activity	nuclear pore	intracellular protein transport
aug_scv1_leest_g7216	mitochondrial carrier		cell	
aug_scv1_leest_g7255	vesicular-fusion protein SEC17		cell;intracellular	transport;protein transport
aug_scv1_leest_g7343	phospholipid-translocating ATPase	binding;transporter activity;nucleotide binding;hydrolase	cell	

		activity		
aug_scv1_leest_g7418	vesicle-mediated transport-related protein			protein transport
aug_scv1_leest_g760	MFS general substrate transporter	transporter activity	cell	transport
aug_scv1_leest_g7622	archain 1		cytoplasm;protein complex	transport;protein transport
aug_scv1_leest_g7656	coatomer protein			transport
aug_scv1_leest_g7686	peptide transporter PTR2A			
aug_scv1_leest_g7744	anion exchanging protein	transporter activity	cell	ion transport
aug_scv1_leest_g7757	ARM repeat-containing protein	transporter activity	nuclear envelope;protein complex	protein transport
aug_scv1_leest_g7859	t-SNARE	protein binding	cell;intracellular	transport;protein transport
aug_scv1_leest_g8004	DnaJ domain-containing protein	protein binding		
aug_scv1_leest_g8015	oligopeptide transporter	binding		transport
aug_scv1_leest_g8157	xanthine/uracil permease			
aug_scv1_leest_g8232	Cl-channel protein	ion channel activity	cell	transport;ion transport
aug_scv1_leest_g8261	MATE efflux family protein	transporter activity	cell	transport
aug_scv1_leest_g8284	GATA transcription factor e	binding;sequence-specific DNA binding transcription factor activity;protein binding		regulation of biological process
aug_scv1_leest_g8300	MFS general substrate transporter		integral to membrane	transmembrane transport
aug_scv1_leest_g8301	related to monocarboxylate transporter 2		integral to membrane	transmembrane transport
aug_scv1_leest_g8339	clathrin heavy chain 1	structural molecule	cytoplasm;protein complex;plasma	transport;protein transport

		activity	membrane;cytoplasmic membrane-bounded vesicle;Golgi apparatus	
aug_scv1_leest_g849	exocyst complex protein		cytoplasm	transport
aug_scv1_leest_g8493	ran/spi1 binding protein	protein binding	cytoplasm	protein metabolic process;catabolic process;protein transport;transport;cell cycle
aug_scv1_leest_g853	ammonium transporter	transporter activity	cell	
aug_scv1_leest_g8767	potassium:hydrogen antiporter			transport
aug_scv1_leest_g880	Arsenite efflux transporter ArsB-like, puative			transport
aug_scv1_leest_g881	MFS general substrate transporter		cell	transport
aug_scv1_leest_g8837	putative Na(+)/H(+) antiporter C3A11,09	transporter activity	cell	ion transport;transport
aug_scv1_leest_g8841	SDA1-domain-containing protein			
aug_scv1_leest_g8850	multidrug resistance-associated ABC transporter	hydrolase activity		
aug_scv1_leest_g8866	mitochondrial carrier protein RIM2		integral to membrane;membrane	transmembrane transport;transport
aug_scv1_leest_g8895	transport protein particle complex subunit		Golgi apparatus;organelle	transport
aug_scv1_leest_g8961	PNTB-domain-containing protein	catalytic activity;nucleotide	cell	ion transport;metabolic process

		binding		
aug_scv1_leest_g8971	signal recognition particle protein	RNA binding;hydrolase activity;nucleotide binding	cytoplasm	nucleobase-containing compound metabolic process;catabolic process;protein transport
aug_scv1_leest_g9024	aminophospholipid-transporting P-type ATPase	binding;hydrolase activity		transport
aug_scv1_leest_g9208	heavy metal translocatin	binding;catalytic activity		ion transport
aug_scv1_leest_g9269	signal recognition particle binding protein	binding;nucleotide binding;hydrolase activity	endoplasmic reticulum;protein complex	nucleobase-containing compound metabolic process;catabolic process;protein transport
aug_scv1_leest_g930	Arsenite efflux transporter ArsB-like, puative			transport
aug_scv1_leest_g9317	quinate permease	transporter activity	cell	transport
aug_scv1_leest_g939	magnesium transporter	transporter activity	cell	ion transport;transport
aug_scv1_leest_g9480	H+/nucleoside cotransporter	transporter activity	cell	transport
aug_scv1_leest_g9501	mitochondrial half-size ABC transporter	hydrolase activity;nucleotide binding		biological_process;transport
aug_scv1_leest_g9506	nuclear pore complex protein		protein complex;nuclear envelope	transport
aug_scv1_leest_g96	coatomer protein	structural molecule activity	Golgi apparatus;protein complex;cytoplasmic membrane-bounded vesicle	transport;protein transport
aug_scv1_leest_g9627	urea transporter	transporter activity	cell	transport
aug_scv1_leest_g9700	importin alpha re-exporter	transporter activity	protein complex;nuclear envelope	protein transport
aug_scv1_leest_g9900	iron-sulfur clusters transporter ATM1	hydrolase activity;transporter activity;nucleotide	cell	transport;nucleobase-containing compound metabolic process;catabolic process

		binding		
aug_scv1_leest_g9920	hexose transporter	transporter activity	cell	transport
aug_scv1_leest_g9932	potassium transporter			
aug_scv1_leest_g9946	ATP synthase d subunit	transporter activity	protein complex;mitochondrion	generation of precursor metabolites and energy;nucleobase-containing compound metabolic process;ion transport;biosynthetic process
aug_scv1_leest_g9949	exocyst complex component sec8			
aug_scv1_leest_g9970	t-SNARE		cell	transport

Supplementary Table S7 Gene set enriched in single-organism cellular process in Group I specific SNPs

Gene ID	Description	Molecule function	Cellular component	Biologic process
aug_scv1_leest_g10002	ion transport domain-containing protein	ion channel activity	cell	ion transport;transport
aug_scv1_leest_g10003	MFS general substrate transporter		cell	transport
aug_scv1_leest_g10073	related to Mitochondrial DnaJ chaperone	protein binding		
aug_scv1_leest_g1019	AousoA		cytoplasm;cell	transport
aug_scv1_leest_g10215	asparaginyl-tRNA synthetase	catalytic activity;nucleic acid binding;nucleotide binding	cytoplasm	nucleobase-containing compound metabolic process;translation
aug_scv1_leest_g1023	transcription factor Tfb2		nucleus	regulation of biological process;response to stress;DNA metabolic process
aug_scv1_leest_g103	dynamin GTPase	nucleotide binding;hydrolase activity		nucleobase-containing compound metabolic process;catabolic process
aug_scv1_leest_g10371	multidrug resistance protein 4		cell	transport
aug_scv1_leest_g10392	vacuolar assembling/sorting protein VPS16		cytoplasm	intracellular protein transport
aug_scv1_leest_g10448	phosphatidyl synthase	transferase activity	cell	biosynthetic process;lipid metabolic process
aug_scv1_leest_g10529	chorismate synthase	catalytic activity		biosynthetic process
aug_scv1_leest_g10552	carbamoyl-phosphate synthase	binding;nucleotide binding;transferase activity		catabolic process;nucleobase-containing compound metabolic process;biosynthetic process
aug_scv1_leest_g10556	beta-tubulin 1 tubb1	hydrolase activity;structural molecule activity;nucleotide binding	cytoskeleton;protein complex;cytoplasm	cellular component organization;nucleobase-containing compound metabolic

				process;catabolic process;biological_process
aug_scv1_leest_g10617	phosphoserine aminotransferase	binding;transferase activity		biosynthetic process
aug_scv1_leest_g10627	glycosyltransferase family 4 protein	transferase activity		carbohydrate metabolic process;biosynthetic process
aug_scv1_leest_g10650	cap			cellular component organization
aug_scv1_leest_g10665	Crm1-F1	transporter activity	protein complex;nuclear envelope	protein transport
aug_scv1_leest_g10708	1,3-beta-glucan synthase	transferase activity	protein complex;plasma membrane	biosynthetic process;carbohydrate metabolic process
aug_scv1_leest_g10713	Ca-transporting ATPase	hydrolase activity;transporter activity;binding;nucleotide binding	cell	ion transport;nucleobase-containing compound metabolic process;biosynthetic process
aug_scv1_leest_g10719	actin-related protein Arp3	nucleotide binding;actin binding	cytoskeleton	regulation of biological process;cytoskeleton organization
aug_scv1_leest_g10737	karyopherin Kap95	transporter activity	protein complex;nuclear envelope	protein transport
aug_scv1_leest_g10903	adenylate cyclase	catalytic activity		biological_process
aug_scv1_leest_g10968	translation initiation factor eIF3 subunit			
aug_scv1_leest_g11002	OPT oligopeptide transporter			transmembrane transport
aug_scv1_leest_g11015	alpha,alpha-trehalase	calcium ion binding;hydrolase activity	cytoplasm	carbohydrate metabolic process;catabolic process
aug_scv1_leest_g11100	histone-lysine N-methyltransferase	transferase activity	nucleus	organelle organization;cellular protein modification process
aug_scv1_leest_g11145	CDF-like metal transporter	transporter activity	cell	ion transport;transport
aug_scv1_leest_g11146	PREDICTED: Werner syndrome	nucleic acid binding;exonuclease	intracellular;nucleus	nucleobase-containing compound

	ATP-dependent helicase homolog	activity;3'-5' exonuclease activity;protein binding		metabolic process
aug_scv1_leest_g11209	ABC transporter	hydrolase activity;nucleotide binding		biological_process;transport
aug_scv1_leest_g11245	eukaryotic translation initiation factor 2 subunit alpha	translation factor activity, nucleic acid binding	cytoplasm;protein complex	translation
aug_scv1_leest_g11288	Protein cwh43		cell	
aug_scv1_leest_g11305	RecName: Full=Flap endonuclease 1; Short=FEN-1; AltName: Full=Flap structure-specific endonuclease 1	nuclease activity;DNA binding;binding	mitochondrion;nucleolus;nucleoplasm	response to stress;DNA metabolic process;biosynthetic process;catabolic process
aug_scv1_leest_g11309	protoheme IX farnesyltransferase	transferase activity	cell	
aug_scv1_leest_g11350	ribonucleotide reductase small subunit	binding;catalytic activity	cytoplasm;protein complex	nucleobase-containing compound metabolic process;metabolic process
aug_scv1_leest_g11384	sodium/hydrogen exchanger	transporter activity	cell	transport;biological_process;ion transport
aug_scv1_leest_g11388	snare-like protein	transferase activity	organelle;intracellular;cytoplasm;cell	cellular component organization;transport;organelle organization
aug_scv1_leest_g11408	MFS general substrate transporter			
aug_scv1_leest_g11410	MFS general substrate transporter		cell	transport
aug_scv1_leest_g11435	elongation factor G mitochondrial	translation factor activity, nucleic acid binding;hydrolase activity;nucleotide binding	mitochondrion	translation;nucleobase-containing compound metabolic process;catabolic process
aug_scv1_leest_g11443	MFS general substrate transporter		cell	transport
aug_scv1_leest_g11447	OPT oligopeptide transporter			transport
aug_scv1_leest_g11486	ras GEF			signal transduction
aug_scv1_leest_g11534	glycoside hydrolase family 10 protein	hydrolase activity		carbohydrate metabolic process

aug_scv1_leest_g11579	methylenetetrahydrofolate reduct	catalytic activity		
aug_scv1_leest_g11625	nime/cyclinb			cell cycle
aug_scv1_leest_g11627	ATP-dependent protease La	peptidase activity		
aug_scv1_leest_g11629	g2/mitotic-specific cyclin cdc13			regulation of biological process;cell cycle
aug_scv1_leest_g11646	vacuolar protein sorting-associated protein vps17	lipid binding	cell;protein complex	cell communication
aug_scv1_leest_g11650	homoserine O-acetyltransferase	transferase activity	cytoplasm	biosynthetic process
aug_scv1_leest_g11683	inositol polyphosphate phosphatase	hydrolase activity		lipid metabolic process
aug_scv1_leest_g11722	FAD-binding protein	catalytic activity;nucleotide binding		biosynthetic process;metabolic process
aug_scv1_leest_g11767	gamma-adaptin	transporter activity	Golgi apparatus;cytoplasm;protein complex	transport;protein transport
aug_scv1_leest_g1178	CNH-domain-containing protein	enzyme regulator activity;lipid binding	intracellular	signal transduction
aug_scv1_leest_g1179	Sec1-like snare protein			
aug_scv1_leest_g11805	MVP1 domain-containing protein	binding		
aug_scv1_leest_g11811	atypical/PIKK/ATM protein kinase			
aug_scv1_leest_g11817	coatomer complex protein	structural molecule activity	cytoplasmic membrane-bounded vesicle;Golgi apparatus;protein complex	transport
aug_scv1_leest_g11829	CDF-like metal transporter	transporter activity	cell	ion transport;transport
aug_scv1_leest_g11839	VHS domain-containing protein		intracellular	protein transport
aug_scv1_leest_g11848	ketol-acid reductoisomerase	catalytic activity;binding		biosynthetic process;metabolic process
aug_scv1_leest_g11916	core histone H2A/H2B/H3/H4 family	DNA binding;protein binding	chromosome;nucleus	organelle organization

	protein			
aug_scv1_leest_g11947	delta 9-fatty acid desaturase	catalytic activity;binding	cell;endoplasmic reticulum	biosynthetic process;lipid metabolic process;metabolic process
aug_scv1_leest_g11964	voltage-dependent ion-selective channel	ion channel activity	mitochondrion	transport;regulation of biological process;ion transport
aug_scv1_leest_g12009	membrane transporter		integral to membrane	transmembrane transport
aug_scv1_leest_g12121	RabGAP TBC			signal transduction
aug_scv1_leest_g12162	fatty acid synthase			
aug_scv1_leest_g12163	diaphanous GTPase-binding domain-containing protein	protein binding		
aug_scv1_leest_g12189	dihydroorotate dehydrogenase	catalytic activity		nucleobase-containing compound metabolic process
aug_scv1_leest_g12202	metalloprotease			
aug_scv1_leest_g12203	metalloprotease	binding;peptidase activity		biosynthetic process;lipid metabolic process;protein metabolic process;catabolic process
aug_scv1_leest_g12253	MFS general substrate transporter	transporter activity	cell	transport
aug_scv1_leest_g12272	lysine-tRNA ligase	nucleic acid binding;catalytic activity;nucleotide binding	cytoplasm	nucleobase-containing compound metabolic process;translation
aug_scv1_leest_g1228	histone cluster 1, H4k	DNA binding	chromosome;nucleus	organelle organization
aug_scv1_leest_g12282	ATP phosphoribosyltransferase	transferase activity		
aug_scv1_leest_g12296	RecName: Full=Protein rad9; AltName: Full=SCC2 homolog			
aug_scv1_leest_g12299	putative mfs transporter protein		cell	transport
aug_scv1_leest_g12367	DNA helicase	hydrolase activity		DNA metabolic process

aug_scv1_leest_g12381	thiamin biosynthesis protein	phosphomethylpyrimidine kinase activity;kinase activity		thiamine biosynthetic process;phosphorylation
aug_scv1_leest_g12419	FAD/NAD(P)-binding domain-containing protein	catalytic activity		
aug_scv1_leest_g12420	kynureninase	hydrolase activity		nucleobase-containing compound metabolic process;biosynthetic process;secondary metabolic process
aug_scv1_leest_g12432	DNA repair-related protein	metal ion binding;zinc ion binding		
aug_scv1_leest_g1244	amino acid transporter	transporter activity	cell	transport
aug_scv1_leest_g12455	GTPase-activating protein gyp7	enzyme regulator activity	intracellular	signal transduction
aug_scv1_leest_g12462	histone H1	DNA binding	chromosome;nucleus	organelle organization
aug_scv1_leest_g12463	related to Beta3 protein (Ruby)		cytoplasm;protein complex	transport;protein transport
aug_scv1_leest_g12488	anthranilate synthase component	catalytic activity		biosynthetic process
aug_scv1_leest_g12521	cyclin-like protein	protein binding	nucleoplasm;protein complex	regulation of biological process;metabolic process;cell cycle
aug_scv1_leest_g12547	methylenetetrahydrofolate reduct	catalytic activity	mitochondrion	metabolic process
aug_scv1_leest_g12575	5-methyltetrahydropteroyltriglutamate-homocysteine S-methyltransferase	binding;transferase activity		biosynthetic process;metabolic process
aug_scv1_leest_g12612	putative peripheral membrane protein required for the formation of cytosolic vesicles			catabolic process;protein transport
aug_scv1_leest_g1264	amidophosphoribosyltransferase	binding;transferase activity		biosynthetic process;nucleobase-containing compound metabolic process
aug_scv1_leest_g13222	rho GTPase activating protein 22	guanyl-nucleotide exchange factor	intracellular	regulation of small GTPase

		activity;GTPase activator activity;phospholipid binding		mediated signal transduction;small GTPase mediated signal transduction;signal transduction;positive regulation of GTPase activity
aug_scv1_leest_g1440	chromosome condensation complex protein		nucleus	organelle organization;cell cycle;biological_process
aug_scv1_leest_g1443	related to Intersectin 1	enzyme regulator activity;lipid binding;actin binding;calcium ion binding	intracellular	signal transduction
aug_scv1_leest_g1454	condensin complex subunit SMC1	nucleotide binding	chromosome;nucleus	organelle organization;cell cycle;DNA metabolic process;response to stress
aug_scv1_leest_g1505	voltage-gated potassium channel beta-2 subunit	ion channel activity	cell	ion transport;transport
aug_scv1_leest_g1509	MFS general substrate transporter		cell	transport
aug_scv1_leest_g1580	transport protein particle complex subunit		intracellular	transport
aug_scv1_leest_g1608	kinesin heavy chain	nucleotide binding;motor activity	cytoskeleton;protein complex	biological_process
aug_scv1_leest_g1613	kinase-like protein	transferase activity;kinase activity		lipid metabolic process;signal transduction
aug_scv1_leest_g1614	glycosyltransferase family 15 protein	transferase activity	cell	biosynthetic process;cellular protein modification process;carbohydrate metabolic process
aug_scv1_leest_g1621	phenylalanyl-tRNA synthetase	catalytic activity;nucleotide binding;binding;RNA binding	cytoplasm	translation;nucleobase-containing compound metabolic process
aug_scv1_leest_g1634	DNA repair helicase	DNA binding;hydrolase activity;nucleotide	nucleus	response to stress;DNA metabolic

		binding		process
aug_scv1_leest_g1657	putative protein PB2B2,06c	hydrolase activity		nucleobase-containing compound metabolic process;catabolic process
aug_scv1_leest_g1668	terpenoid synthase	terpene synthase activity;magnesium ion binding;lyase activity		
aug_scv1_leest_g1759	MutS protein homolog 4	nucleotide binding;DNA binding		response to stress;DNA metabolic process
aug_scv1_leest_g1762	vacuolar sorting-associated protein Vps27	binding	intracellular	protein transport
aug_scv1_leest_g1789	cystathionine beta-lyase	catalytic activity;binding;kinase activity;nucleotide binding	cytoplasm	biosynthetic process;metabolic process;lipid metabolic process
aug_scv1_leest_g1821	Adaptor protein complex AP-3 delta subunit	transporter activity	cytoplasm;protein complex;Golgi apparatus;cell;endosome	protein transport;transport;biological_process;cell differentiation;regulation of biological process;multicellular organismal development;cellular component organization
aug_scv1_leest_g1832	chloroplast lipoate protein ligase	catalytic activity;transferase activity	cytoplasm	cellular protein modification process;biosynthetic process
aug_scv1_leest_g1876	mitochondrial alternative oxidase	binding;catalytic activity	cell;mitochondrion	biological_process;generation of precursor metabolites and energy
aug_scv1_leest_g1923	60S acidic ribosomal protein P2	structural molecule activity	ribosome	translation
aug_scv1_leest_g1961	acetate--CoA ligase	nucleotide binding;catalytic activity		metabolic process
aug_scv1_leest_g1995	regulator of filamentous growth and virulence Rfg1	DNA binding;sequence-specific DNA binding transcription factor activity		symbiosis, encompassing mutualism through parasitism;response to biotic stimulus;response to external

				stimulus;cell communication;response to stress;regulation of biological process
aug_scv1_leest_g2035	Sec1-like protein			transport
aug_scv1_leest_g2106	enolase C-terminal domain-like protein	catalytic activity		catabolic process
aug_scv1_leest_g2138	coatomer subunit alpha-2	structural molecule activity	Golgi apparatus;protein complex;cytoplasmic membrane-bounded vesicle	transport;protein transport
aug_scv1_leest_g2150	RecF/RecN/SMC protein	nucleotide binding	organelle;intracellular	DNA metabolic process;organelle organization
aug_scv1_leest_g2165	translation initiation factor 6	translation factor activity, nucleic acid binding;binding		translation;organelle organization
aug_scv1_leest_g2206	aspartate kinase	kinase activity		biosynthetic process;metabolic process
aug_scv1_leest_g2223	NADH kinase	kinase activity		metabolic process
aug_scv1_leest_g2279	Fph type histidine kinase	protein kinase activity;signal transducer activity		primary metabolic process;metabolic process;signal transduction
aug_scv1_leest_g2308	RecName: Full=Ras-like protein; Flags: Precursor	hydrolase activity;nucleotide binding	plasma membrane	signal transduction;nucleobase-containin g compound metabolic process;catabolic process
aug_scv1_leest_g2335	tars protein	catalytic activity;nucleotide binding	cytoplasm	nucleobase-containing compound metabolic process;translation
aug_scv1_leest_g2358	RecName: Full=Double-strand break repair protein MRE11	nuclease activity;binding	nucleus	cell cycle;response to stress;DNA metabolic process

aug_scv1_leest_g2396	sec24-like protein			transport;protein transport
aug_scv1_leest_g2424	ATP binding protein			transport
aug_scv1_leest_g2429	isocitrate lyase	catalytic activity		metabolic process
aug_scv1_leest_g2433	pantothenate kinase	nucleotide binding;kinase activity		nucleobase-containing compound metabolic process;biosynthetic process;metabolic process
aug_scv1_leest_g2449	DASH complex subunit dam1 domain-containing protein			
aug_scv1_leest_g245	OPT oligopeptide transporter	transporter activity		transport
aug_scv1_leest_g246	OPT oligopeptide transporter	transporter activity		transport
aug_scv1_leest_g2460	methionyl-tRNA synthetase	nucleotide binding;catalytic activity	cytoplasm	nucleobase-containing compound metabolic process;translation
aug_scv1_leest_g2463	1-phosphatidylinositol-3-phosphate 5-kinase	binding;kinase activity		lipid metabolic process;metabolic process
aug_scv1_leest_g2496	u5 small nuclear ribonucleoprotein component	nucleic acid binding;calcium ion binding;hydrolase activity;protein binding;nucleotide binding	intracellular;endoplasmic reticulum	protein metabolic process;nucleobase-containing compound metabolic process;catabolic process
aug_scv1_leest_g2528	branched-chain-amino-acid aminotransferase			biosynthetic process
aug_scv1_leest_g2529	aconitate hydratase	catalytic activity;binding		generation of precursor metabolites and energy;catabolic process
aug_scv1_leest_g2530	aconitate hydratase	catalytic activity;binding		generation of precursor metabolites and energy;catabolic process
aug_scv1_leest_g2610	unnamed protein product	transporter activity	cell	transport

aug_scv1_leest_g2633	Mis12-domain-containing protein		chromosome;nucleus	organelle organization;cell cycle
aug_scv1_leest_g2643	adrenoleukodystrophy protein	hydrolase activity;nucleotide binding	cell	transport;nucleobase-containing compound metabolic process;catabolic process
aug_scv1_leest_g2648	ubiquinol-cytochrome c reductase iron-sulfur subunit	binding;catalytic activity;transporter activity	cell;mitochondrion	generation of precursor metabolites and energy
aug_scv1_leest_g2724	aromatic-L-amino-acid decarboxylase	binding;catalytic activity		
aug_scv1_leest_g2763	anthranilate synthase component II	catalytic activity		biosynthetic process
aug_scv1_leest_g2810	general substrate transporter	transporter activity	cell	transport
aug_scv1_leest_g2814	TPR-like protein		nucleus;protein complex	regulation of biological process;organelle organization;cell cycle;biological_process
aug_scv1_leest_g2815	Dbl domain-containing protein	enzyme regulator activity		
aug_scv1_leest_g285	PAS domain-containing protein	signal transducer activity		signal transduction
aug_scv1_leest_g2914	P-type ATPase	binding;hydrolase activity;transporter activity;nucleotide binding	cell	nucleobase-containing compound metabolic process;catabolic process;ion transport
aug_scv1_leest_g2980	isoleucine-tRNA ligase	catalytic activity;nucleotide binding	cytoplasm	nucleobase-containing compound metabolic process;translation;regulation of biological process
aug_scv1_leest_g2982	related to Cutl1 or CASP protein		Golgi apparatus	transport
aug_scv1_leest_g3052	eukaryotic translation initiation factor 2A	translation factor activity, nucleic acid binding		translation
aug_scv1_leest_g3057	expressed protein		protein complex;nuclear envelope	transport

aug_scv1_leest_g3058	RabGAP/TBC	enzyme regulator activity	intracellular	signal transduction
aug_scv1_leest_g3086	septin	lipid binding;structural molecule activity;nucleotide binding	cell;cytoskeleton;protein complex;cell wall;cytoplasm	cell cycle;metabolic process;regulation of biological process
aug_scv1_leest_g3248	phenylalanine ammonia-lyase	catalytic activity	cytoplasm	secondary metabolic process;biosynthetic process;catabolic process
aug_scv1_leest_g3262	ATP18 subunit J of the mitochondrial F1F0 ATP synthase	transporter activity	intracellular;protein complex	generation of precursor metabolites and energy;nucleobase-containing compound metabolic process;ion transport;biosynthetic process
aug_scv1_leest_g3279	uridine 5'-monophosphate synthase/orotate phosphoribosyltransferase	transferase activity		nucleobase-containing compound metabolic process
aug_scv1_leest_g3318	APC amino acid permease	transporter activity	cell	transport
aug_scv1_leest_g3393	homoserine kinase	nucleotide binding;kinase activity		metabolic process
aug_scv1_leest_g3424	dynein heavy chain protein 1	motor activity;nucleotide binding;hydrolase activity	cytoskeleton;protein complex	nucleobase-containing compound metabolic process;catabolic process;biological_process
aug_scv1_leest_g3471	6,7-dimetjyl-8-ribityllumazine synthase	transferase activity	cell;protein complex	biosynthetic process
aug_scv1_leest_g367	DNA glycosylase	nucleic acid binding;catalytic activity		response to stress;DNA metabolic process
aug_scv1_leest_g3739	histone-fold-containing protein	DNA binding	chromosome;nucleus	organelle organization
aug_scv1_leest_g3742	L-methionine transporter	transporter activity	cell	transport
aug_scv1_leest_g3914	mitochondrial NAD transporter		cell	transport
aug_scv1_leest_g3942	Major facilitator superfamily		cell	transport
aug_scv1_leest_g4041	alpha/beta hydrolase family	hydrolase activity	endoplasmic reticulum	lipid metabolic process;protein

	domain-containing protein			transport
aug_scv1_leest_g4060	MFS general substrate transporter		cell	transport
aug_scv1_leest_g4077	f-box protein pof6		cytoplasm	transport
aug_scv1_leest_g4153	APC amino acid permease		cell	transport
aug_scv1_leest_g4179	GTP cyclohydrolase I	hydrolase activity	cytoplasm	biosynthetic process
aug_scv1_leest_g418	phosphatidylinositol phosphate kinase PIPK5			metabolic process
aug_scv1_leest_g4229	acetolactate synthase	nucleotide binding;binding;transferase activity	mitochondrion	biosynthetic process
aug_scv1_leest_g425	nuclear membrane protein			biological_process
aug_scv1_leest_g4279	4-hydroxybenzoate polyprenyl transferase	transferase activity	cell	biosynthetic process
aug_scv1_leest_g4296	DNA ligase	nucleotide binding;binding;catalytic activity		response to stress;DNA metabolic process
aug_scv1_leest_g4380	EF-hand	enzyme regulator activity;kinase activity;calcium ion binding	Golgi apparatus	metabolic process;signal transduction;biological_process
aug_scv1_leest_g4391	MFS general substrate transporter		cell	transport
aug_scv1_leest_g4489	phosphoglycerate kinase	kinase activity;nucleotide binding	cytoplasm	metabolic process;generation of precursor metabolites and energy;carbohydrate metabolic process;catabolic process
aug_scv1_leest_g4502	COQ7 protein	binding;catalytic activity		biosynthetic process;metabolic process
aug_scv1_leest_g4559	SDHC, cytochrome b subunit of succinate dehydrogenase	catalytic activity;electron carrier activity	intracellular;protein complex	generation of precursor metabolites and energy;catabolic process
aug_scv1_leest_g4580	malate dehydrogenase	nucleotide binding;binding;catalytic activity		metabolic process

aug_scv1_leest_g4595	uncharacterized protein	zinc ion binding		
aug_scv1_leest_g4603	F-actin capping protein, alpha subunit	actin binding	F-actin capping protein complex	actin cytoskeleton organization
aug_scv1_leest_g4616	other/TTK protein kinase	nucleotide binding;protein kinase activity		cellular protein modification process
aug_scv1_leest_g4617	xylanase	hydrolase activity;carbohydrate binding	extracellular region	carbohydrate metabolic process;catabolic process
aug_scv1_leest_g4631	phospho-2-dehydro-3-deoxyheptonate aldolase	transferase activity		biosynthetic process
aug_scv1_leest_g464	saccharopine dehydrogenase	nucleotide binding		
aug_scv1_leest_g4673	amino acid transporter		cell	transport
aug_scv1_leest_g4685	P-loop containing nucleoside triphosphate hydrolase protein	hydrolase activity;transporter activity;nucleotide binding	cell	transport;nucleobase-containing compound metabolic process;catabolic process
aug_scv1_leest_g4696	cell division control protein 54	hydrolase activity;DNA binding;nucleotide binding	protein complex;nuclear chromosome;nucleoplasm	biological_process;DNA metabolic process;biosynthetic process
aug_scv1_leest_g4725	pleiotropic drug resistance ABC transporter	hydrolase activity;transporter activity;nucleotide binding	cell	transport;nucleobase-containing compound metabolic process;catabolic process
aug_scv1_leest_g4731	putative hexose transporter protein	transporter activity	cell	transport
aug_scv1_leest_g4748	ATP synthase E chain domain-containing protein	transporter activity	protein complex;mitochondrion	generation of precursor metabolites and energy;nucleobase-containing compound metabolic process;ion transport;biosynthetic process
aug_scv1_leest_g4755	microtubule associated protein	microtubule binding		cytokinesis

aug_scv1_leest_g487	initiation factor 3	translation factor activity, nucleic acid binding;binding	protein complex;cytoplasm;nucleus	regulation of biological process;translation
aug_scv1_leest_g4876	serine/threonine-protein phosphatase 2B catalytic subunit A1	phosphoprotein phosphatase activity	intracellular;protein complex	cellular component organization;reproduction;signal transduction;cellular homeostasis
aug_scv1_leest_g4887	gamma-glutamyltranspeptidase	transferase activity		
aug_scv1_leest_g4904	mitochondrial carrier		cell	transport
aug_scv1_leest_g4993	3-hydroxyisobutyrate dehydrogenase	nucleotide binding;catalytic activity		nucleobase-containing compound metabolic process;carbohydrate metabolic process;catabolic process;secondary metabolic process
aug_scv1_leest_g5028	tubulin gamma chain	hydrolase activity;nucleotide binding	cytoskeleton;protein complex;microtubule organizing center	cellular component organization;nucleobase-containing compound metabolic process;catabolic process;biological_process
aug_scv1_leest_g5043	DNA helicase	DNA binding;hydrolase activity;nucleotide binding		response to stress;DNA metabolic process
aug_scv1_leest_g5071	ubiquitin-conjugating enzyme E2	nucleotide binding;catalytic activity		
aug_scv1_leest_g512	GTP-binding protein ypt5	nucleotide binding		protein transport;signal transduction
aug_scv1_leest_g5156	tyrosine tRNA ligase	catalytic activity;nucleotide binding	cytoplasm	nucleobase-containing compound metabolic process;translation
aug_scv1_leest_g516	cyclin-like protein		nucleoplasm;protein complex	regulation of biological process;primary metabolic process;cell cycle;metabolic process

aug_scv1_leest_g5171	dumpy, isoform O			
aug_scv1_leest_g5202	voltage-gated potassium channel beta-2 subunit	ion channel activity	cell	ion transport;transport
aug_scv1_leest_g5215	tyrosine protein phosphatase	phosphoprotein phosphatase activity		
aug_scv1_leest_g5243	histidine kinase	transcription regulator activity;signal transducer activity;nucleotide binding;protein kinase activity	cell;intracellular	signal transduction;cellular protein modification process;regulation of biological process
aug_scv1_leest_g5266	oxidase, peroxisomal	catalytic activity		lipid metabolic process;metabolic process
aug_scv1_leest_g5352	signal transducer	binding		signal transduction
aug_scv1_leest_g5384	delta-1-pyrroline-5-carboxylate dehydrogenase	catalytic activity	mitochondrion	metabolic process;catabolic process;biosynthetic process
aug_scv1_leest_g5398	3-hydroxybutyryl-CoA dehydrogenase	binding;nucleotide binding;catalytic activity		
aug_scv1_leest_g5458	glutathione reductase	nucleotide binding;catalytic activity;antioxidant activity	cytoplasm	regulation of biological process;cellular homeostasis;metabolic process
aug_scv1_leest_g5544	OPT oligopeptide transporter			transport
aug_scv1_leest_g560	MFS general substrate transporter		cell	transport
aug_scv1_leest_g5659	carbohydrate esterase family 1 protein	hydrolase activity	cytoplasmic membrane-bounded vesicle	
aug_scv1_leest_g5675	mitochondrial amino-acid acetyltransferase	transferase activity	mitochondrion	biosynthetic process
aug_scv1_leest_g5702	DigA protein	binding	intracellular	transport;protein transport
aug_scv1_leest_g5739	gamma-tubulin ring complex protein		microtubule organizing center;cytoskeleton	cytoskeleton organization
aug_scv1_leest_g5766	transthyretin			transport

aug_scv1_leest_g5780	dihydroxy-acid dehydratase	catalytic activity		biosynthetic process
aug_scv1_leest_g5783	septin family protein, P-loop GTPase	nucleotide binding		cell cycle
aug_scv1_leest_g5841	DUF1692-domain-containing protein			
aug_scv1_leest_g5872	Ndufs1, NADH-ubiquinone oxidoreductase 75kD subunit (775)	electron carrier activity;binding;catalytic activity	cell	generation of precursor metabolites and energy
aug_scv1_leest_g5926	Setd1a protein	nucleic acid binding;transferase activity;nucleotide binding	nucleus	organelle organization;cellular protein modification process
aug_scv1_leest_g5955	NAD-specific glutamate dehydrogenase	catalytic activity;nucleotide binding		catabolic process;metabolic process
aug_scv1_leest_g5963	transmembrane GTPase fzo1	hydrolase activity;nucleotide binding	cell	nucleobase-containing compound metabolic process;catabolic process
aug_scv1_leest_g5991	MFS general substrate transporter		cell	transport
aug_scv1_leest_g6000	kinesin domain-containing protein	nucleotide binding;motor activity	cytoskeleton;protein complex	biological_process
aug_scv1_leest_g6002	white collar photoreceptors-like protein	receptor activity;sequence-specific DNA binding transcription factor activity;DNA binding;signal transducer activity;binding		signal transduction;regulation of biological process
aug_scv1_leest_g6028	MFS general substrate transporter	transporter activity	cell	transport
aug_scv1_leest_g6047	DNA repair protein	DNA binding;hydrolase activity;nucleotide binding	nucleus	lipid metabolic process;response to stress;DNA metabolic process
aug_scv1_leest_g6056	histone deacetylase complex, catalytic component RPD3	hydrolase activity	nucleus	regulation of biological process;cellular protein modification process;organelle organization
aug_scv1_leest_g6057	GTP-binding protein TypA	hydrolase activity;nucleotide binding	intracellular	nucleobase-containing compound metabolic process;catabolic process

aug_scv1_leest_g6094	ATPase V1 complex subunit C, partial	hydrolase activity;transporter activity	cell;protein complex	ion transport
aug_scv1_leest_g6161	MFS general substrate transporter		cell	transport
aug_scv1_leest_g6173	activator of Hsp90 ATPase	ATPase activator activity;chaperone binding		positive regulation of ATPase activity;response to stress
aug_scv1_leest_g6179	MFS general substrate transporter		cell	transport
aug_scv1_leest_g6232	Acireductone dioxygenase	catalytic activity	nucleus	biosynthetic process;metabolic process
aug_scv1_leest_g6275	oligopeptide transporter			transport
aug_scv1_leest_g6307	general substrate transporter	transporter activity	cell	transport
aug_scv1_leest_g6346	tryptophanyl-tRNA synthetase	catalytic activity;nucleotide binding		translation;nucleobase-containing compound metabolic process
aug_scv1_leest_g6380	putative alpha-ketoglutarate-dependent taurine dioxygenase protein	catalytic activity		metabolic process
aug_scv1_leest_g6432	endoplasmic reticulum-derived transport vesicle ERV46			transport
aug_scv1_leest_g6433	vacuolar protein sorting-associated protein 45			transport
aug_scv1_leest_g6439	cell division control protein 25			
aug_scv1_leest_g6487	CoaE-domain-containing protein	kinase activity;nucleotide binding		biosynthetic process;nucleobase-containing compound metabolic process
aug_scv1_leest_g6488	isoleucyl-tRNA synthetase	nucleotide binding;catalytic activity		translation;nucleobase-containing compound metabolic process
aug_scv1_leest_g6508	DNA binding protein	nucleotide binding;DNA binding	nucleus	response to stress;DNA metabolic process
aug_scv1_leest_g663	gamma-tubulin complex DGRIP91/SPC98 component		microtubule organizing center;cytoskeleton	cytoskeleton organization

aug_scv1_leest_g669	Adenylosuccinate synthetase	catalytic activity;nucleotide binding;binding	cytoplasm	biosynthetic process;nucleobase-containing compound metabolic process
aug_scv1_leest_g6743	uncharacterized protein		intracellular	protein transport
aug_scv1_leest_g6747	phosphoribosylformylglycinamidin	catalytic activity		biosynthetic process;nucleobase-containing compound metabolic process
aug_scv1_leest_g6753	3-deoxy-7-phosphoheptulonate synthase	transferase activity		biosynthetic process
aug_scv1_leest_g6828	MFS general substrate transporter		cell	transport
aug_scv1_leest_g6844	related to lactose permease	transporter activity	cell	transport
aug_scv1_leest_g6928	indole-3-glycerol phosphate synthase	catalytic activity;transferase activity		biosynthetic process
aug_scv1_leest_g6929	TFIIH basal transcription factor complex subunit SSL1	binding	nucleus	response to stress;DNA metabolic process;regulation of biological process
aug_scv1_leest_g6969	tyrosine-tRNA ligase	nucleotide binding;catalytic activity		translation;nucleobase-containing compound metabolic process
aug_scv1_leest_g6983	RabGAP/TBC	enzyme regulator activity	intracellular	signal transduction
aug_scv1_leest_g7013	CPII coat sec24 protein	binding	cytoplasmic membrane-bounded vesicle;Golgi apparatus;protein complex	protein transport;transport
aug_scv1_leest_g7016	serine-tRNA ligase	nucleotide binding;catalytic activity	cytoplasm	nucleobase-containing compound metabolic process;translation
aug_scv1_leest_g7057	eukaryotic translation initiation factor 3 subunit A	translation initiation factor activity	cytoplasm	translational initiation;translation
aug_scv1_leest_g7063	Rad51-associated protein Brh2			response to stress;DNA metabolic process

aug_scv1_leest_g7065	PhoX domain-containing protein			cell communication
aug_scv1_leest_g7066	Gcd10p-domain-containing protein	translation factor activity, nucleic acid binding		translation
aug_scv1_leest_g707	HIS4	catalytic activity;nucleotide binding;hydrolase activity;binding		biosynthetic process;metabolic process
aug_scv1_leest_g7086	V-snare-domain-containing protein		Golgi apparatus;cell	transport;protein transport
aug_scv1_leest_g7178	gamma-glutamyltranspeptidase	transferase activity		
aug_scv1_leest_g7193	ribulose-5-phosphate 3-epimerase	catalytic activity;binding		carbohydrate metabolic process;nucleobase-containing compound metabolic process;catabolic process;secondary metabolic process
aug_scv1_leest_g7226	UROD/MetE-like protein	5-methyltetrahydropteroyltriglutamate-homocysteine S-methyltransferase activity		methionine biosynthetic process
aug_scv1_leest_g7230	imidazoleglycerol phosphate synthase	catalytic activity;transferase activity	cytoplasm	biosynthetic process
aug_scv1_leest_g7255	vesicular-fusion protein SEC17		cell;intracellular	transport;protein transport
aug_scv1_leest_g7284	DNA ligase I	DNA binding;nucleotide binding;catalytic activity		DNA metabolic process;biosynthetic process;response to stress
aug_scv1_leest_g7289	Isopentenyldiphosphate isomerase	hydrolase activity;catalytic activity		biosynthetic process;lipid metabolic process
aug_scv1_leest_g7387	NdufA5, NADH-ubiquinone oxidoreductase ETC complex subunit	catalytic activity	mitochondrion	generation of precursor metabolites and energy
aug_scv1_leest_g7397	aspartate-tRNA ligase	nucleic acid binding;nucleotide binding;catalytic activity	cytoplasm	nucleobase-containing compound metabolic process;translation
aug_scv1_leest_g7415	elongation factor Tu	translation factor activity, nucleic acid	intracellular	translation;nucleobase-containing

		binding;hydrolase activity;nucleotide binding		compound metabolic process;catabolic process
aug_scv1_leest_g7418	vesicle-mediated transport-related protein			protein transport
aug_scv1_leest_g7458	dihydrolipoamide acetyltransferase	transferase activity	cytoplasm;protein complex	metabolic process
aug_scv1_leest_g7479	mitochondrial Lipoyl synthase	binding;transferase activity	mitochondrion	biosynthetic process;cellular protein modification process
aug_scv1_leest_g7483	SNF2 family DNA-dependent ATPase	nucleic acid binding;hydrolase activity;nucleotide binding		response to stress
aug_scv1_leest_g7501	P-loop containing nucleoside triphosphate hydrolase protein	ribosylnicotinamide kinase activity		nicotinamide nucleotide biosynthetic process
aug_scv1_leest_g7512	RhoGAP-domain-containing protein		cytoskeleton	signal transduction
aug_scv1_leest_g760	MFS general substrate transporter	transporter activity	cell	transport
aug_scv1_leest_g7622	archain 1		cytoplasm;protein complex	transport;protein transport
aug_scv1_leest_g7641	ribose-5-phosphate isomerase	catalytic activity	cytoplasm	nucleobase-containing compound metabolic process;carbohydrate metabolic process;catabolic process;secondary metabolic process
aug_scv1_leest_g7642	aromatic amino acid family biosynthesis-like protein	catalytic activity;kinase activity;transferase activity;binding;nucleotide binding	cytoplasm	metabolic process;biosynthetic process
aug_scv1_leest_g7649	Diphosphomevalonate decarboxylase	catalytic activity;nucleotide binding		biosynthetic process;lipid metabolic process
aug_scv1_leest_g7656	coatomer protein			transport
aug_scv1_leest_g7678	delta 9-fatty acid desaturase protein	catalytic activity;binding	cell;endoplasmic reticulum	biosynthetic process;lipid metabolic process;metabolic process
aug_scv1_leest_g7741	urease accessory protein UreG	binding;molecular_function;nucleotide	cytoplasm	nucleobase-containing compound

		binding;hydrolase activity;protein binding		metabolic process;catabolic process
aug_scv1_leest_g7757	ARM repeat-containing protein	transporter activity	nuclear envelope;protein complex	protein transport
aug_scv1_leest_g7839	homoserine O-acetyltransferase	transferase activity	cytoplasm	biosynthetic process
aug_scv1_leest_g7847	GTP-binding protein 1	nucleotide binding		
aug_scv1_leest_g785	phenylalanine-tRNA ligase	catalytic activity;nucleotide binding;RNA binding	cytoplasm	translation;nucleobase-containing compound metabolic process
aug_scv1_leest_g7851	iron-sulphar subunit protein of succinate dehydrogenase	catalytic activity;electron carrier activity;binding		generation of precursor metabolites and energy;catabolic process
aug_scv1_leest_g7859	t-SNARE	protein binding	cell;intracellular	transport;protein transport
aug_scv1_leest_g789	FolC bifunctional protein	catalytic activity		biosynthetic process
aug_scv1_leest_g7926	TBP-domain-containing protein	DNA binding;translation factor activity, nucleic acid binding		translation;cellular component organization
aug_scv1_leest_g7929	VpsA protein	hydrolase activity;nucleotide binding		nucleobase-containing compound metabolic process;catabolic process
aug_scv1_leest_g7943	uracil-DNA glycosylase	hydrolase activity		response to stress;DNA metabolic process
aug_scv1_leest_g796	RIM15, signal transduction response regulator	protein kinase activity;signal transducer activity;nucleotide binding		primary metabolic process;metabolic process;signal transduction
aug_scv1_leest_g7997	PIN domain-like protein	nuclease activity;DNA binding	nucleus	response to stress;DNA metabolic process
aug_scv1_leest_g7998	oligosaccharyl transferase STT3 subunit	transferase activity	cell	biosynthetic process;cellular protein modification

				process;carbohydrate metabolic process
aug_scv1_leest_g8004	DnaJ domain-containing protein	protein binding		
aug_scv1_leest_g8015	oligopeptide transporter	binding		transport
aug_scv1_leest_g8033	uridine/cytidine kinase	kinase activity;transferase activity;nucleotide binding		nucleobase-containing compound metabolic process;biosynthetic process;metabolic process
aug_scv1_leest_g8102	DNA polymerase lambda	DNA-directed DNA polymerase activity;DNA binding		nucleotide-excision repair
aug_scv1_leest_g8157	xanthine/uracil permease			
aug_scv1_leest_g822	SWR1-complex protein 4			biological_process
aug_scv1_leest_g8232	Cl-channel protein	ion channel activity	cell	transport;ion transport
aug_scv1_leest_g8261	MATE efflux family protein	transporter activity	cell	transport
aug_scv1_leest_g828	tubulin alpha	hydrolase activity;structural molecule activity;nucleotide binding	cytoskeleton;protein complex;cytoplasm	cellular component organization;nucleobase-containin g compound metabolic process;catabolic process;biological_process
aug_scv1_leest_g8284	GATA transcription factor e	binding;sequence-specific DNA binding transcription factor activity;protein binding		regulation of biological process
aug_scv1_leest_g8291	gamma-glutamyl phosphate reductase	nucleotide binding;catalytic activity		biosynthetic process;metabolic process
aug_scv1_leest_g8300	MFS general substrate transporter		integral to membrane	transmembrane transport
aug_scv1_leest_g8301	related to monocarboxylate transporter 2		integral to membrane	transmembrane transport
aug_scv1_leest_g8316	barren			
aug_scv1_leest_g8333	sulfite reductase subunit beta	nucleotide binding;binding;catalytic activity	intracellular;protein complex	biosynthetic process;metabolic process

aug_scv1_leest_g8339	clathrin heavy chain 1	structural molecule activity	cytoplasm;protein complex;plasma membrane;cytoplasmic membrane-bounded vesicle;Golgi apparatus	transport;protein transport
aug_scv1_leest_g8372	signal transducer	lipid binding		regulation of biological process;cell communication
aug_scv1_leest_g844	nicotinate-nucleotide diphosphorylase	transferase activity		biosynthetic process;nucleobase-containing compound metabolic process;secondary metabolic process
aug_scv1_leest_g8451	aspartate kinase homoserine dehydrogenase	nucleotide binding;kinase activity;catalytic activity		metabolic process
aug_scv1_leest_g849	exocyst complex protein		cytoplasm	transport
aug_scv1_leest_g8493	ran/spi1 binding protein	protein binding	cytoplasm	protein metabolic process;catabolic process;protein transport;transport;cell cycle
aug_scv1_leest_g8495	kinesin-like protein	nucleotide binding;motor activity	cytoskeleton;protein complex	biological_process
aug_scv1_leest_g8505	5-aminolevulinate synthase	transferase activity;binding	mitochondrion	biosynthetic process
aug_scv1_leest_g853	ammonium transporter	transporter activity	cell	
aug_scv1_leest_g8560	guanylate kinase	kinase activity		metabolic process;nucleobase-containing compound metabolic process
aug_scv1_leest_g8577	glycoside hydrolase family 13 protein	hydrolase activity;binding		generation of precursor metabolites and energy;biosynthetic process;carbohydrate metabolic

				process
aug_scv1_leest_g8647	thymidylate synthase	transferase activity		metabolic process;nucleobase-containing compound metabolic process;biosynthetic process
aug_scv1_leest_g8683	histidine kinase			
aug_scv1_leest_g8761	RasGAP domain-containing protein			regulation of biological process
aug_scv1_leest_g8799	mitochondrial genome maintenance protein		mitochondrion;cytoplasmic chromosome	mitochondrion organization
aug_scv1_leest_g881	MFS general substrate transporter		cell	transport
aug_scv1_leest_g8834	RecName: Full=UMP-CMP kinase; AltName: Full=Deoxycytidylate kinase; Short=CK; Short=dCMP kinase; AltName: Full=Uridine monophosphate/cytidine monophosphate kinase; Short=UMP/CMP kinase; Short=UMP/CMPK	kinase activity;nucleotide binding	cytoplasm	nucleobase-containing compound metabolic process;biosynthetic process
aug_scv1_leest_g8837	putative Na(+)/H(+) antiporter C3A11,09	transporter activity	cell	ion transport;transport
aug_scv1_leest_g8841	SDA1-domain-containing protein			
aug_scv1_leest_g8866	mitochondrial carrier protein RIM2		integral to membrane;membrane	transmembrane transport;transport
aug_scv1_leest_g8895	transport protein particle complex subunit		Golgi apparatus;organelle	transport
aug_scv1_leest_g8897	mitochondrial chaperone BCS1	nucleotide binding		
aug_scv1_leest_g8908	DNA-(apurinic or apyrimidinic site) lyase	nuclease activity;DNA binding	intracellular	response to stress;DNA metabolic process
aug_scv1_leest_g8925	siroheme synthase	catalytic activity;nucleotide binding		biosynthetic process;metabolic process
aug_scv1_leest_g8930	DNA glycosylase	catalytic activity		response to stress;DNA metabolic

				process
aug_scv1_leest_g8971	signal recognition particle protein	RNA binding;hydrolase activity;nucleotide binding	cytoplasm	nucleobase-containing compound metabolic process;catabolic process;protein transport
aug_scv1_leest_g9037	unnamed protein product, partial	protein binding;enzyme regulator activity;kinase activity	intracellular;protein complex;cytoplasm;nucleus	cell cycle;metabolic process;regulation of biological process;organelle organization
aug_scv1_leest_g9092	ras-domain-containing protein	nucleotide binding		
aug_scv1_leest_g914	WD40 repeat-like protein			metabolic process;regulation of biological process
aug_scv1_leest_g9142	argininosuccinate synthetase	nucleotide binding;catalytic activity		biosynthetic process
aug_scv1_leest_g9150	GTP cyclohydrolase II	binding;catalytic activity;hydrolase activity		biosynthetic process
aug_scv1_leest_g9174	ThrRS/AlaRS common domain-containing protein	nucleotide binding		
aug_scv1_leest_g9188	DS-domain-containing protein			cellular protein modification process
aug_scv1_leest_g9190	sec7 guanine nucleotide exchange factor	enzyme regulator activity	intracellular	signal transduction
aug_scv1_leest_g9206	ras GTPase-activating protein	enzyme regulator activity		biological_process;signal transduction
aug_scv1_leest_g9221	Phosphoribosylformimino-5-aminoimidazole carboxamide ribotide isomerase	catalytic activity		biosynthetic process
aug_scv1_leest_g9254	PX domain-containing protein	phosphatidylinositol binding		cell communication
aug_scv1_leest_g9266	Metallo-hydrolase/oxidoreductase	hydrolase activity;binding		biosynthetic process
aug_scv1_leest_g9269	signal recognition particle binding protein	binding;nucleotide binding;hydrolase activity	endoplasmic reticulum;protein complex	nucleobase-containing compound metabolic process;catabolic process;protein transport
aug_scv1_leest_g9308	translation initiation factor IF-2	translation factor activity, nucleic acid	intracellular	translation;nucleobase-containing

		binding;hydrolase activity;nucleotide binding		compound metabolic process;catabolic process
aug_scv1_leest_g932	arginine biosynthesis bifunctional protein ARG7	transferase activity	mitochondrion	biosynthetic process
aug_scv1_leest_g9336	glucan synthesis regulatory protein			biological_process
aug_scv1_leest_g9355	phosphatidylserine decarboxylase	calcium ion binding;catalytic activity		biosynthetic process;lipid metabolic process
aug_scv1_leest_g939	magnesium transporter	transporter activity	cell	ion transport;transport
aug_scv1_leest_g94	C4-methyl sterol oxidase	catalytic activity;binding	cell	biosynthetic process;lipid metabolic process;metabolic process
aug_scv1_leest_g9501	mitochondrial half-size ABC transporter	hydrolase activity;nucleotide binding		biological_process;transport
aug_scv1_leest_g9509	eukaryotic translation initiation factor 3 subunit 8	translation factor activity, nucleic acid binding	protein complex;cytoplasm	translation
aug_scv1_leest_g9552	probable methionyl-tRNA synthetase, mitochondrial	ATP binding;methionine-tRNA ligase activity;aminoacyl-tRNA ligase activity;nucleotide binding;ligase activity	cytoplasm	methionyl-tRNA aminoacylation;tRNA aminoacylation for protein translation
aug_scv1_leest_g9587	XPG I-region protein	nuclease activity		DNA repair;nucleic acid phosphodiester bond hydrolysis
aug_scv1_leest_g9597	TBC-domain-containing protein	enzyme regulator activity;calcium ion binding	intracellular	signal transduction
aug_scv1_leest_g9599	condensin complex subunit SMC2			DNA metabolic process;organelle organization
aug_scv1_leest_g96	coatomer protein	structural molecule activity	Golgi apparatus;protein complex;cytoplasmic membrane-bounded vesicle	transport;protein transport

aug_scv1_leest_g9681	kinesin-like protein			
aug_scv1_leest_g9683	GTP-binding protein	nucleotide binding	intracellular	signal transduction
aug_scv1_leest_g9684	Eukaryotic translation initiation factor 5B Short=eIF-5B	nucleotide binding;translation factor activity, nucleic acid binding;binding;hydrolase activity	ribosome;cytosol;mitochondrion	nucleobase-containing compound metabolic process;catabolic process;regulation of biological process;translation
aug_scv1_leest_g9700	importin alpha re-exporter	transporter activity	protein complex;nuclear envelope	protein transport
aug_scv1_leest_g9710	methylated-DNA--cysteine S-met	catalytic activity;methylated-DNA-[protein]-cysteine S-methyltransferase activity		DNA repair
aug_scv1_leest_g9714	NAD-malate dehydrogenase	catalytic activity;nucleotide binding		carbohydrate metabolic process;metabolic process;generation of precursor metabolites and energy;catabolic process
aug_scv1_leest_g9732	mannosyltransferase 1	transferase activity		
aug_scv1_leest_g9734	glutamine amidotransferase subunit pdxT	transferase activity		glutamine metabolic process
aug_scv1_leest_g979	L-asparaginase	hydrolase activity		
aug_scv1_leest_g981	Guf1 protein	hydrolase activity;nucleotide binding	mitochondrion	translation;nucleobase-containing compound metabolic process;catabolic process
aug_scv1_leest_g9855	valine-tRNA ligase	catalytic activity;nucleotide binding	cytoplasm	nucleobase-containing compound metabolic process;translation;regulation of biological process
aug_scv1_leest_g9866	acetylglutamate kinase	transferase activity;binding	mitochondrion	biosynthetic process

aug_scv1_leest_g9900	iron-sulfur clusters transporter ATM1	hydrolase activity;transporter activity;nucleotide binding	cell	transport;nucleobase-containing compound metabolic process;catabolic process
aug_scv1_leest_g9932	potassium transporter			
aug_scv1_leest_g9946	ATP synthase d subunit	transporter activity	protein complex;mitochondrion	generation of precursor metabolites and energy;nucleobase-containing compound metabolic process;ion transport;biosynthetic process
aug_scv1_leest_g9967	aminoimidazole ribonucleotide synthetase	binding;nucleotide binding;catalytic activity	cytoplasm	nucleobase-containing compound metabolic process;biosynthetic process
aug_scv1_leest_g9991	seryl-tRNA synthetase	catalytic activity;nucleotide binding		nucleobase-containing compound metabolic process;translation

Supplementary Table S8 Gene set enriched in methylation in Group III specific SNPs

Gene ID	Description	Molecule function	Cellular component	Biologic process
aug_scv1_leest_g10488	putative methyltransferase	transferase activity;RNA binding	nucleus	nucleobase-containing compound metabolic process
aug_scv1_leest_g10508	Diphthine synthase	transferase activity		cellular protein modification process;biosynthetic process;metabolic process
aug_scv1_leest_g11100	histone-lysine N-methyltransferase	transferase activity	nucleus	organelle organization;cellular protein modification process
aug_scv1_leest_g12127	protein arginine N-methyltransferase	transferase activity	cytoplasm	cellular protein modification process
aug_scv1_leest_g12425	NOL1/NOP2/sun family RNA met	transferase activity;RNA binding		nucleobase-containing compound metabolic process;metabolic process
aug_scv1_leest_g2477	Rsm22-domain-containing protein	transferase activity		metabolic process;translation
aug_scv1_leest_g2985	S-adenosyl-L-methionine-dependent methyltransferase	transferase activity		
aug_scv1_leest_g4571	ubiquinone menaquinone biosynthesis methyltransferase UbiE	transferase activity		metabolic process
aug_scv1_leest_g5926	Setd1a protein	nucleic acid binding;transferase activity;nucleotide binding	nucleus	organelle organization;cellular protein modification process
aug_scv1_leest_g6315	histone methyltransferase	transferase activity	chromosome;nucleus	regulation of biological process;cellular protein modification process;organelle organization

aug_scv1_leest_g6351	rRNA adenine dimethylase	transferase activity		nucleobase-containing compound metabolic process
aug_scv1_leest_g7084	tRNA (guanine-N(1)-)-methyltransferase	transferase activity	mitochondrion	metabolic process
aug_scv1_leest_g742	ICMT-domain-containing protein	transferase activity		metabolic process
aug_scv1_leest_g7447	FtsJ-domain-containing protein	transferase activity		metabolic process;nucleobase-containing compound metabolic process
aug_scv1_leest_g7627	sphingolipid C9-methyltransferase			lipid biosynthetic process
aug_scv1_leest_g7648	delta-sterol C-methyltransferase	transferase activity		biosynthetic process;lipid metabolic process;metabolic process
aug_scv1_leest_g7918	tRNA guanosine-2'-O-methyltransferase	nucleic acid binding;methyltransferase activity		methylation
aug_scv1_leest_g8230	S-adenosyl-L-methionine-dependent methyltransferase	transferase activity		metabolic process
aug_scv1_leest_g8437	tRNA methyltransferase	transferase activity		metabolic process;nucleobase-containing compound metabolic process
aug_scv1_leest_g8647	thymidylate synthase	transferase activity		metabolic process;nucleobase-containing compound metabolic process;biosynthetic process
aug_scv1_leest_g8843	nucleolar essential protein 1	transferase activity		metabolic process
aug_scv1_leest_g9798	ICMT-domain-containing protein		cell	

Supplementary Table S9 Range, maximum, minimum, mean, standard error (SE), and coefficients of variation (CV) for the 13 phenotypic traits of *Lentinula edodes*

Traits	Range	Minimum	Maximum	Mean	SE	CV
FP	107	183	76	118	4.47	27.88%
FB	104	196	92	1348	4.56	24.98%
NF	184	186	2	25	4.23	122.66%
Y	581.66	603.46	21.8	193.82	19.06	72.27%
WF	33	34.86	1.86	13.438	1.34	73.29%
DGR-myg	3.68	6.26	2.58	4.37	0.15	26.03%
DGR-sd	1.83	4.35	2.52	3.63	0.06	12.55%
PW	30.08	31.46	1.38	9.69	0.85	64.87%
SW	15.25	15.76	0.51	4.29	0.4	68.09%
PD	45.73	70.34	24.61	43.94	1.27	21.19%
PT	13.47	17.39	3.92	9.74	0.42	31.35%
SD	11.97	17.46	5.49	10.44	0.43	30.55%
SL	46.54	64.93	18.39	41.11	1.47	26.21%

Supplementary Discussion S1

Under stress conditions, effective signal transduction is a premise that passes environmental information into the inside of a fungal cell. There are several signaling pathways which have evolved in fungi to respond to environmental stresses, including MAPK (mitogen activated protein kinase), cAMP-PKA (cyclic AMP-protein kinase A), TOR (target-of-rapamycin), and Ca^{2+}-calcineurin pathways[1,2]. Gene aug_scv1_leest_g2962 codes for a Pbs2-like MAPKK protein. In fungi, the Hog1-mediated MAPK pathway is widely involved in resistance to thermal shocks, osmotic stress, oxidative stress and heavy metals[1]. Pbs2 is responsible for activating Hog1 in the Hog1 pathway[3]. Recently, the Hog1 pathway has been reported to be involved in stress response in macro-basidiomycetes, *Heterobasidion annosum*[4]. Gene aug_scv1_leest_g2964, encoding a kinase similar to *Saccharomyces cerevisiae* Yak1. Yak1 phosphorylates and activates the transcription factors Hsf1 (heat-shock transcription factor) and Msn2, which play important roles in cellular homeostasis by activating gene expression during stress conditions, including heat shock, nutrient starvation and oxidative stress[5]. Yak1, along with phosphoprotein Pop2, functions as a part of a glucose-sensing system that controls the growth of yeast[6]. Hsf1 is conserved from yeast to humans and it is essential for the viability of an organism[7] and it is also a master regulator of HSP (heat-shock protein) expression[8].

Fungal heat-shock proteins (HSP) also function as crucial regulators of stress response, and are involved in various biological pathways. Aug_scv1_leest_g6029 codes for a homolog of Hsp40, also called DnaJ. Hsp40 is a cofactor of the Hsp70 (DnaK) responsible for the initial folding of nascent polypeptide in biotic and abiotic stresses[9,10]. In bacteria, the DnaK/DnaJ/GrpE chaperone functions as a critical thermosensor detecting temperature variation by modulating conformational changes of GrpE[11].

Characteristics of lipid in membrane is another factor that contributes to stress response[12]. In mushroom-forming fungi, membrane alteration is a stress signal that triggers the shift from vegetative growth to reproductive growth[13]. The homologs of several genes involved in lipid metabolism have been identified here. Aug_scv1_leest_g3811 encodes a glycerol-3-phosphate o-acyltransferase. In bacteria, acyltransferases control the temperature-dependent remodeling of membrane lipid A under different environmental temperature conditions[14]. Aug_scv1_leest_g4530 codes for a C4-methyl sterol oxidase that participates in the fatty acid biosynthetic process. Ergosterol biosynthesis is the pathway responsible for the construction of ergosterol – an important component of fungal membrane – and it is required for maintaining the membrane stability and fluidity[15]. In *Aspergillus fumigatus*, the C4-methyl sterol oxidase is involved in maintaining canonical ergosterol biosynthesis and the stress response to the environment[16].

Twelve other genes are related to stress response and fruiting body initiation and they are involved in the population differentiation of one or two groups (Table 2). Aug_scv1_leest_g5779 and aug_scv1_leest_g8857, code for PKC-like kinase. Gene aug_scv1_leest_g6391 encodes tuberin and gene aug_scv1_leest_g7437 codes for guanine nucleotide-exchange factor 1 (GEF1). The homologs of these genes were reported to serve in stress-signaling pathways[17-20]. A homolog of BAG domain-containing protein encoding by aug_scv1_leest_g2961, which can interact with and regulate Hsp70 under stress conditions[21].

Aug_scv1_leest_g4052 codes for a platelet-activating factor acetylhydrolase 1 (PAC1), and aug_scv1_leest_g9682 encodes cyclopropane fatty acid synthase (*cfs1*), which take part in fatty acid metabolism during stress response in fungi[13,22].

Transcription factors (TFs) govern changes in gene expression in response to environmental stresses. PriB TF encoded by aug_scv1_leest_g8832, is related to fruiting body initiation in *L. edodes*[23]. A GATA transcription factor encoded by aug_scv1_leest_g9618, whose homolog acts as modulator of stress response[24]. Aug_scv1_leest_g3008 codes for endoplasmic reticulum oxidoreductin 1, and aug_scv1_leest_g7483 encodes a SNF2 family DNA-dependent ATPase, whose homologs interact with Hsf1 and respond to temperature stress in

yeast[25,26].

In addition, gene productions of aug_scv1_leest_g9807 and aug_scv1_leest_g12501 are DEAD box RNA helicases, which are key factors in the cold response in fungi[27].

References

1. Bahn, Y.S. *et al.* Sensing the environment: lessons from fungi. *Nat. Rev. Microbiol.* **5**, 57-69 (2007).
2. Alonso-Monge, R., Román, E., Arana, D., Pla, J. & Nombela, C. Fungi sensing environmental stress. *Clin. Microbiol. Infec.* **15**, 17-19 (2009).
3. Arana, D. M., Nombela, C., Alonso-Monge, R. & Pla, J. The Pbs2 MAP kinase kinase is essential for the oxidative-stress response in the fungal pathogen *Candida albicans*. *Microbiology* **151**, 1033-1049 (2005).
4. Raffaello, T., Keriö, S. & Asiegbu, F. O. Role of the HaHOG1 MAP kinase in response of the conifer root and but rot pathogen (*Heterobasidion annosum*) to osmotic and oxidative stress. *PLoS ONE* **7**, e31186 (2012).
5. Lee, P., Cho, B. R., Joo, H. S. & Hahn, J. S. Yeast Yak1 kinase, a bridge between PKA and stress-responsive transcription factors, Hsf1 and Msn2/Msn4. *Mol. Microbiol.* **70**, 882-895 (2008).
6. Moriya, H. *et al.* Yak1p, a DYRK family kinase, translocates to the nucleus and phosphorylates yeast Pop2p in response to a glucose signal. *Gene. Dev.* **15**, 1217-1228 (2001).
7. Brown, A. J. *et al.* Stress adaptation in a pathogenic fungus. *J. Exp. Biol.* **217**, 144-155 (2014).
8. Fujimoto, M. Transcriptional regulation by HSF. In *Heat Shock Factor* 73-89 (Springer, 2016).
9. Burnie, J. P., Carter, T. L., Hodgetts, S. J. & Matthews, R. C. Fungal heat-shock proteins in human disease. *FEMS Microbiology Reviews* **30**, 53-88 (2006).
10. Tiwari, S., Thakur, R. & Shankar, J. Role of heat-shock proteins in cellular function and in the biology of fungi. *Biotechnology Research International* **2015**, 132635 (2015).
11. Shapiro, R. S. & Cowen, L. E. Thermal control of microbial development and virulence: molecular mechanisms of microbial temperature sensing. *mBio* **3**, e00238-12 (2012).
12. Leach, M. D. & Cowen, L. E. To sense or die: mechanisms of temperature sensing in fungal pathogens. *Current Fungal Infection Reports* **8**, 185-191 (2014).
13. Liu, Y., Srivilai, P., Loos, S., Aebi, M. & Kües, U. An essential gene for fruiting body initiation in the basidiomycete *Coprinopsis cinerea* is homologous to bacterial cyclopropane fatty acid synthase genes. *Genetics* **172**, 873-884 (2006).
14. Li, Y. *et al.* LPS remodeling is an evolved survival strategy for bacteria. *Proc. Natl. Acad. Sci. USA* **109**, 8716-8721 (2012).
15. Van Leeuwen, M., Smant, W., De Boer, W. & Dijksterhuis, J. Filipin is a reliable in situ marker of ergosterol in the plasma membrane of germinating conidia (spores) of *Penicillium discolor* and stains intensively at the site of germ tube formation. *J. Microbiol. Meth.* **74**, 64-73 (2008).
16. Blosser, S. J., Merriman, B., Grahl, N., Chung, D. & Cramer, R. A. Two C4-sterol methyl oxidases (Erg25) catalyse ergosterol intermediate demethylation and impact environmental stress adaptation in *Aspergillus fumigatus*. *Microbiology* **160**, 2492-2506 (2014).
17. Rohde, J., Heitman, J. & Cardenas, M. E. The TOR kinases link nutrient sensing to cell growth. *J. Biol. Chem.* **276**, 9583-9586 (2001).
18. Rossman, K. L., Der, C. J. & Sondek, J. GEF means go: turning on RHO GTPases with guanine nucleotide-exchange factors. *Nat. Rev. Mol. Cell Biol.* **6**, 167-180 (2005).
19. Liu, L. & Parent, C. A. TOR kinase complexes and cell migration. *J. Cell Biol.* **194**, 815-824 (2011).
20. Horiuchi, H. & Katayama, T. Protein kinase C of filamentous fungi and its roles in the stresses affecting

hyphal morphogenesis and conidiation. In *Stress Biology of Yeasts and Fungi* 185-198 (Springer, 2015).

21. Takayama, S. & Reed, J. C. Molecular chaperone targeting and regulation by BAG family proteins. *Nat. Cell Biol.* **3**, E237-E241 (2001).

22. Yu, C. *et al.* Biological role of *Trichoderma harzianum*-derived Platelet-Activating Factor Acetylhydrolase (PAF-AH) on stress response and antagonism. *PLoS ONE* **9**, e100367 (2014).

23. Endo, H., Kajiwara, S., Tsunoka, O. & Shishido, K. A novel cDNA, priBc, encoding a protein with a Zn (II) 2Cys6 zinc cluster DNA-binding motif, derived from the basidiomycete *Lentinus edodes*. *Gene* **139**, 117-121 (1994).

24. Laor, D., Cohen, A., Kupiec, M. & Weisman, R. TORC1 regulates developmental responses to nitrogen stress via regulation of the GATA transcription factor Gaf1. *mBio* **6**, e00959-15 (2015).

25. Takemori, Y., Sakaguchi, A., Matsuda, S., Mizukami, Y. & Sakurai, H. Stress-induced transcription of the endoplasmic reticulum oxidoreductin gene ERO1 in the yeast *Saccharomyces cerevisiae*. *Mol. Genet. Genomics* **275**, 89-96 (2006).

26. Shivaswamy, S. & Iyer, V. R. Stress-dependent dynamics of global chromatin remodeling in yeast: dual role for SWI/SNF in the heat shock stress response. *Mol. Cell Biol.* **28**, 2221-2234 (2008).

27. Ellison, C. E. *et al.* Population genomics and local adaptation in wild isolates of a model microbial eukaryote. *Proc. Natl. Acad. Sci. USA* **108**, 2831-2836 (2011).
